# PIP5K1C phosphoinositide kinase deficiency distinguishes PIKFYVE-dependent cancer cells from non-malignant cells

**DOI:** 10.1080/15548627.2023.2182594

**Published:** 2023-03-22

**Authors:** Ajit Roy, Arup R. Chakraborty, Tyzoon Nomanbhoy, Melvin L. DePamphilis

**Affiliations:** a Division of Developmental Biology, National Institute of Child Health & Human Development, National Institutes of Health, Bethesda, MD, USA; b ActivX Biosciences, La Jolla, California, USA

**Keywords:** autophagy, lysosome, PtdIns(4,5)P_2_, PIKFYVE, PIP2, PIP4K2C, PIP5K1C, WX8

## Abstract

Although PIKFYVE phosphoinositide kinase inhibitors can selectively eliminate PIKFYVE-dependent human cancer cells *in vitro* and *in vivo*, the basis for this selectivity has remained elusive. Here we show that the sensitivity of cells to the PIKFYVE inhibitor WX8 is not linked to PIKFYVE expression, macroautophagic/autophagic flux, the BRAF^V600E^ mutation, or ambiguous inhibitor specificity. PIKFYVE dependence results from a deficiency in the PIP5K1C phosphoinositide kinase, an enzyme required for conversion of phosphatidylinositol-4-phosphate (PtdIns4P) into phosphatidylinositol-4,5-bisphosphate (PtdIns[4,5]P_2_/PIP2), a phosphoinositide associated with lysosome homeostasis, endosome trafficking, and autophagy. PtdIns(4,5)P_2_ is produced via two independent pathways. One requires PIP5K1C; the other requires PIKFYVE and PIP4K2C to convert PtdIns3P into PtdIns(4,5)P_2_. In PIKFYVE-dependent cells, low concentrations of WX8 specifically inhibit PIKFYVE *in situ*, thereby increasing the level of its substrate PtdIns3P while suppressing PtdIns(4,5)P_2_ synthesis and inhibiting lysosome function and cell proliferation. At higher concentrations, WX8 inhibits both PIKFYVE and PIP4K2C *in situ*, which amplifies these effects to further disrupt autophagy and induce cell death. WX8 did not alter PtdIns4P levels. Consequently, inhibition of PIP5K1C in WX8-resistant cells transformed them into sensitive cells, and overexpression of PIP5K1C in WX8-sensitive cells increased their resistance to WX8. This discovery suggests that PIKFYVE-dependent cancers could be identified clinically by low levels of PIP5K1C and treated with PIKFYVE inhibitors.

**Abbreviations:** DMSO: dimethylsulfoxide; ELISA: enzyme-linked immunosorbent assay; LC3-I: microtubule associated protein light chain 3-I; LC3-II: microtubule associated protein light chain 3-II; MS: mass spectrometry; PtdIns: phosphatidylinositol; PtdIns3P: PtdIns-3-phosphate; PtdIns4P: PtdIns-4-phosphate; PtdIns5P: PtdIns-5-phosphate; PtdIns(3,5)P_2_: PtdIns-3,5-bisphosphate; PtdIns(4,5)P_2_/PIP2: PtdIns-4,5-bisphosphate; PtdIns(3,4,5)P_3_/PIP3: PtdIns-3,4,5-trisphosphate; PIKFYVE: phosphoinositide kinase, FYVE-type zinc finger containing; PIK3C3: phosphatidylinositol 3-kinase catalytic subunit type 3; PI4KA: phosphatidylinositol 4-kinase alpha; PI4KB: phosphatidylinositol 4-kinase beta; PI4K2A: phosphatidylinositol 4-kinase type 2 alpha; PI4K2B: phosphatidylinositol 4-kinase type 2 beta; PIP4K2A: phosphatidylinositol-5-phosphate 4-kinase type 2 alpha; PIP4K2B: phosphatidylinositol-5-phosphate 4-kinase type 2 beta; PIP4K2C: phosphatidylinositol-5-phosphate 4-kinase type 2 gamma; PIP5K1A: phosphatidylinositol-4-phosphate 5-kinase type 1 alpha; PIP5K1B: phosphatidylinositol-4-phosphate 5-kinase type 1 beta; PIP5K1C: phosphatidylinositol-4-phosphate 5-kinase type 1 gamma; WX8: 1H-indole-3-carbaldehyde (4-anilino-6-[4-morpholinyl]-1,3,5-triazin-2-yl)hydrazone

## Introduction

Inhibition of autophagy as a therapeutic approach to the selective termination of cancer cells has received considerable attention [1,2]. Nevertheless, the role of autophagy in cancer remains paradoxical [3–5]. In nonmalignant cells, autophagy promotes genomic stability by maintaining homeostasis, thereby suppressing cancer, but in malignant cells, autophagy promotes cancer by allowing them to proliferate and migrate under conditions where normal cells become quiescent. Therefore, systemic disruption of autophagy by experimental gene ablation or naturally occurring gene mutations can lead to cancer, whereas pharmacologic interruption of autophagy, either alone or in combination with chemotherapy, can cause tumor regression [4].

Autophagy-dependent cells are recognized in three ways: dependence on one or more autophagy-dependent genes, such as *ATG5* and *ATG7* [6], sensitivity to inhibitors of lysosomal activity such as chloroquine and its derivatives [7], or sensitivity to inhibitors that disrupt lysosome homeostasis, endosomal trafficking, and autophagic flux such as inhibitors of the PIKFYVE phosphoinositide kinase [8–12], an enzyme essential for mammalian embryonic development [13].

To date, efforts to selectively terminate autophagy-dependent cancers have relied on chloroquine and hydroxychloroquine to impair lysosomal activity [2,4,14]. However, interrupting autophagy with these compounds might not be achieved in an acidic tumor microenvironment, because such an environment could neutralize them, thereby allowing cancer cells to survive acidic stress by upregulating autophagy [15,16]. In contrast, PIKFYVE inhibitors rapidly and reversibly disrupt lysosome homeostasis (microautophagy and chaperone-mediated autophagy), endosome trafficking (cellular homeostasis), and fusion of lysosomes with autophagosomes (macroautophagy), thereby effectively suppressing nutrient recovery and energy production in PIKFYVE-dependent cancer cells.

PIKFYVE converts phosphatidylinositol (PtdIns) into phosphatidylinositol-3,5-bisphosphate (PtdIns [3,5]P_2_), a signaling lipid as well as the major precursor for synthesis of phosphatidylinositol-5-phosphate (PtdIns5P) via 3’-dephosphorylation [17]. PtdIns5P, which can also be synthesized directly from PtdIns by PIKFYVE [18], is one of two precursors for synthesis of PtdIns(4,5)P_2_, a signaling lipid in lysosome homeostasis and autophagy [19–21]. Thus, the ability of PIKFYVE inhibitors to selectively terminate autophagy-dependent cancer cells and retard the growth of tumors derived from them suggests that they have therapeutic potential [8–12,22].

PIKFYVE inhibitors can be organized into four groups of chemical analogs that are distinguished by their central core element and adducts (Fig. S1). In cultured cells, they mimic the effects of siRNA or shRNA targeted against *PIKFYVE* gene expression and preferentially suppress PtdIns(3,5)P_2_ synthesis, but in cells bearing specific PIKFYVE mutations, they are ineffective [9,10]. PIKFYVE inhibitors rapidly and reversibly prevent lysosome fission without preventing homotypic lysosome fusion [8], thereby producing enlarged lysosomes visible as cytoplasmic vacuoles and reduced lysosomal activity due to disrupted trafficking of molecules [8], cathepsin maturation [8–10], and intracellular exchange of molecules [23–26]. Thus, PIKFYVE inhibitors inhibit cell proliferation and exhibit therapeutic potential against viruses that require receptor-mediated endocytosis and diseases that trigger expression of pro-inflammatory chemokines by Toll-mediated endocytosis [27].

PIKFYVE inhibitors also disrupt autophagic flux by preventing heterotypic fusion of lysosomes with autophagosomes, thereby exhibiting therapeutic potential against cancers that depend on autophagy for viability [8–12,28]. Taken together with the lysosomal defects, these effects can terminate autophagy-dependent cancer cells under conditions wherein nonmalignant cells remain viable. However, the mechanism that distinguishes autophagy-dependent cancer cells from nonmalignant cells has remained elusive.

To resolve this puzzle, the PIKFYVE inhibitor WX8 was selected as a paradigm for PIKFYVE-specific inhibitors, because it has a low dissociation constant with PIKFYVE *in vitro*, and its secondary target is PIP4K2C (phosphatidylinositol-5-phosphate 4-kinase type 2 gamma) (Fig. S2) [8,10,11]. PIP4K2C is one of three isozymes that act downstream of PIKFYVE to produce phosphatidylinositol-4,5-bisphosphate (PtdIns [4,5]P_2_), a phosphoinositide required for lysosomal homeostasis and heterotypic fusion of lysosomes with autophagosomes [19–21].

The present study was carried out in three phases. The first phase evaluated whether or not the ability of WX8 to distinguish sensitive from resistant human cells was linked to differences in PIKFYVE gene expression, PIKFYVE protein levels, autophagic flux, the presence of the BRAF^V600E^ mutation, or ambiguous inhibitor specificity. No links were found.

The second phase evaluated whether or not inhibition of PIKFYVE had the same effects on cell viability as inhibition of both PIKFYVE and PIP4K2C. The results revealed that selective inhibition of PIKFYVE disrupted lysosome homeostasis and suppressed cell proliferation in both sensitive and resistant cells, but cell death was induced selectively in WX8 sensitive cells when both PIKFYVE and PIP4K2C were inhibited.

The third phase quantified changes in phosphoinositide kinase gene transcripts, phosphoinositide kinase proteins, and phosphoinositide levels in WX8-sensitive and resistant cell lines. The results revealed that the ability of WX8 to selectively terminate autophagy-dependent cancer cells was linked to differences in cellular levels of the enzymes required to produce PtdIns(4,5)P_2_. WX8-sensitive cells were deficient in PIP5K1C, an enzyme required to convert PtdIns4P into PtdIns(4,5)P_2_. Inhibition or ablation of PIP5K1C in WX8-resistant cells converted them into sensitive cells, whereas over-expression of PIP5K1C in WX8-sensitive cells increased their resistance to WX8.

## Results

### WX8 sensitivity was not linked to either PIKFYVE RNA or protein levels

RNA sequence profiling revealed that WX8 neither upregulated nor downregulated *PIKFYVE* RNA either in human melanoma A375 cells or in human foreskin fibroblasts HFF1 (Fig. S3D). The fold changes (Log_2_FC) were less than ±1.5. To determine whether or not sensitivity to WX8 is linked to cellular levels of PIKFYVE protein, 11 different mammalian cell lines representing 4 different cancers and 3 different nonmalignant tissues were cultured under identical conditions in the presence of either the vehicle [dimethyl sulfoxide (DMSO)] or WX8.

Viability refers to the ability of cells to survive and proliferate. Nanomolar concentrations of PIKFYVE inhibitors rapidly and reversibly decrease cell viability, as defined by reduced ATP levels, cell proliferation, and colony formation [8,9]. Therefore, the sensitivity of a cell’s viability to WX8 was quantified by the concentration of WX8 that reduced its ATP level by half (IC_50_).

**Figure 1.**
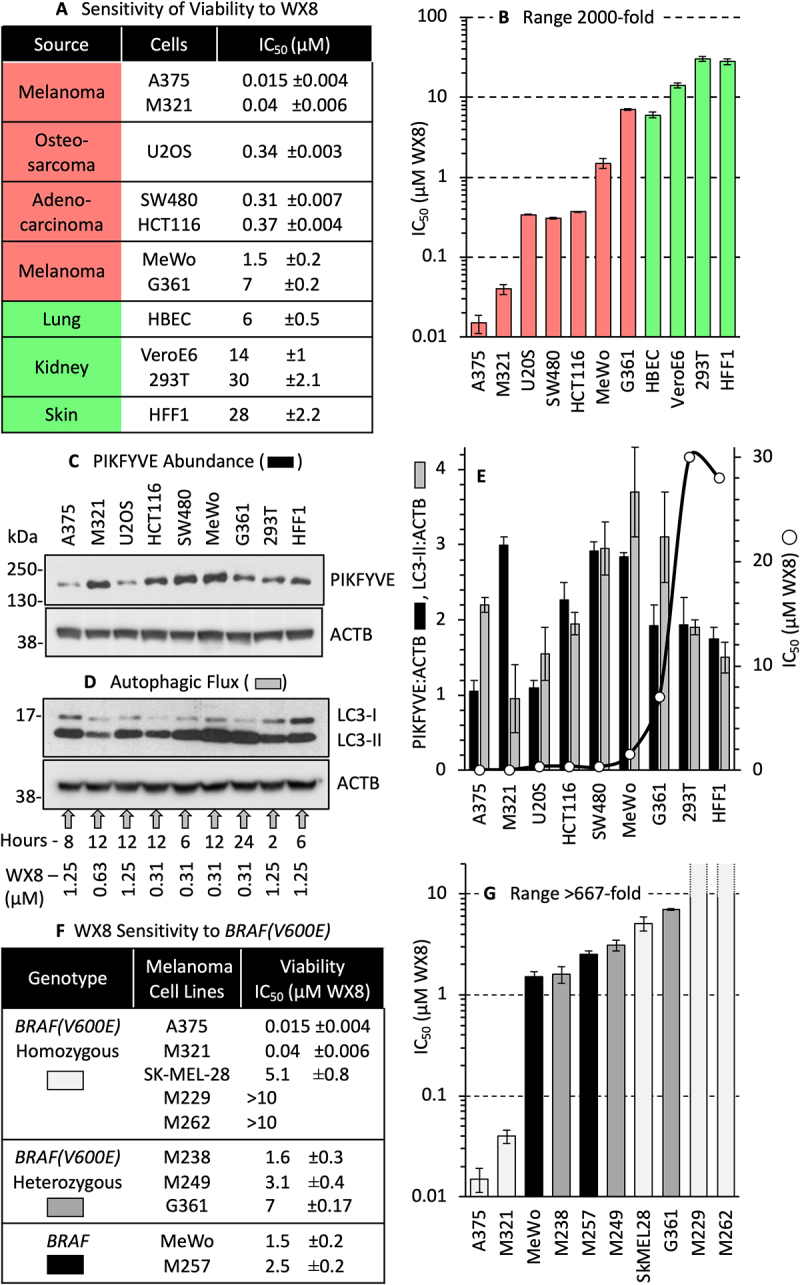
WX8 sensitivity was not linked to PIKFYVE abundance, autophagic flux or the *BRAF^V600E^* mutation. (A) Eleven human cell lines representing three cancer (red) and three normal (green) tissues were cultured with eight different concentrations of WX8 for four days before cellular ATP levels were quantified and their IC_50_ values (µM WX8) calculated ±SEM (three replicates). (B) IC_50_ data from panel A displayed on a logarithmic scale. (C) Nine human cell lines were quantified for PIKFYVE abundance. They represent cells that are highly sensitive (A375, M321), sensitive (U20S, HCT116, SW480), moderately sensitive (MeWo, G361), and resistant (293 T, HFF1) to WX8. (D) The relative capacity for autophagy in each of the cell lines in panel C is displayed by the immunoblot produced (shown here) showing the maximum amount of LC3-II produced when cells were cultured with the optimum concentration of WX8 (step #1) in the time (step #2) required to produce the maximum amount of LC3-II (see Materials and Methods). (E) PIKFYVE:ACTB ratios (black bars) from panel C, and LC3-II:ACTB (gray bars) from panel D were plotted ±SD (two replicates) and compared with cell viability from panel A (WX8 IC_50_ for ATP loss, open circles). (F) IC_50_ values (±SEM, three replicates) for ATP loss with WX8 were determined for 10 different melanoma cell lines [72] with the indicated BRAF genotype. (G) Data in panel F are displayed on a logarithmic scale. Black, gray, and light gray indicate wild-type, heterozygous and homozygous cell lines, respectively.

**Figure 2.**
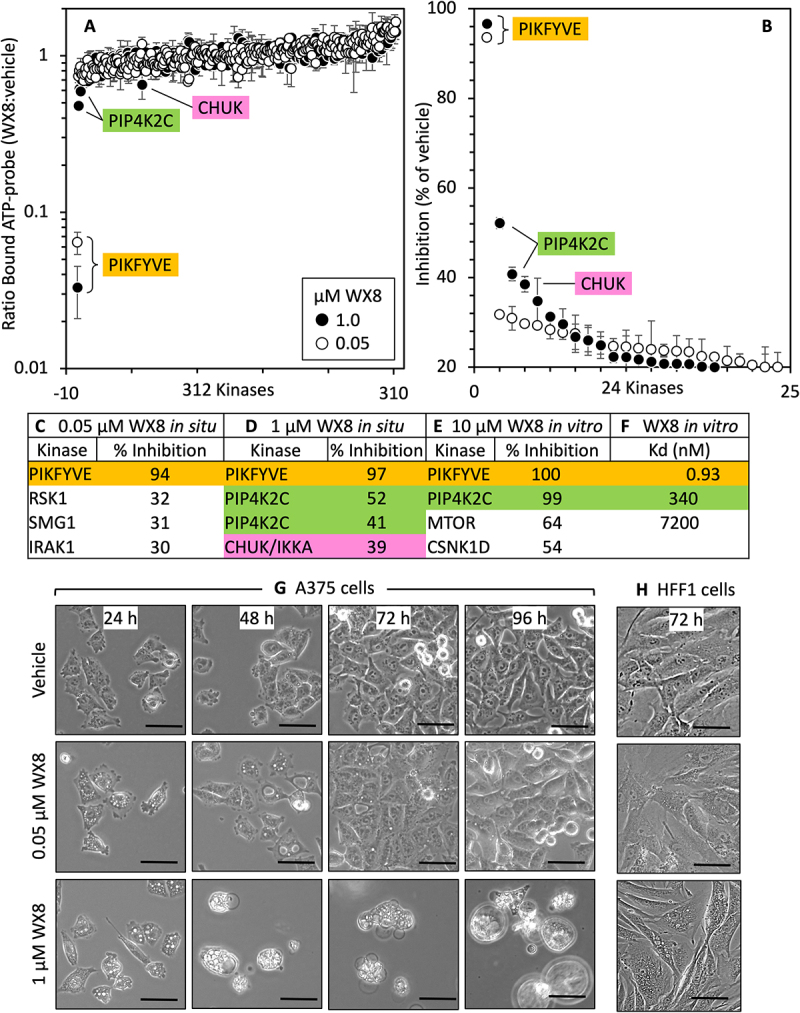
WX8ʹs primary target *in situ* was PIKFYVE and it secondary was PIP4K2C. (A) WX8 was profiled quantitatively against all the 312 kinases detected in melanoma A375 cells under conditions where protein-protein interactions, differential phosphorylation, and other naturally occurring modifications are preserved (ActivX Biosciences). The ratio of the amount of ATP site-specific probe bound to each kinase in WX8 treated cells to the amount bound in vehicle treated cells is plotted. Error bars indicate the standard deviation for three independent assays. The Student T-test score was <0.04. (B) The 24 kinases inhibited 20% or more by WX8 were ordered according to “% of vehicle” (± SEM) and identified for cells cultured with 0.05 µM or 1 µM WX8. Kinases for which inhibition by was <20% are considered nonspecific effects. (C) Kinases inhibited ≥25% *in situ* by 0.05 µM WX8. (D) Kinases inhibited ≥25% *in situ* by 1 µM WX8. In cases where two or more peptides from the same kinase were detected, the greatest inhibition value was used. This value correlated best with orthogonal assays, such as cell-based phosphorylation assays. (E) Kinases inhibited ≥20% *in vitro* by 10 µM WX8 [8]. Percent inhibition compares WX8 treated samples versus control samples. (F) IC_50_ of three top targets determined through *in vitro* kinase binding assay. Figure S7 lists the 24 kinases most sensitive to WX8 inhibition *in situ* (±SEM) and their relative affinity to WX8 *in vitro*. (G) Images of melanoma A375 cells treated with either 0.05 µM or 1 µM WX8 and photographed under phase contrast (scale bar: 50 µm). (H) Images of HFF1 cells treated as in panel G. (scale bar: 20 µm).

Since the sensitivity of cells to WX8 was linked to the density at which cells were seeded (Fig. S4A-C), the effects of WX8 were quantified with cells seeded at less than 5000 cells/cm^2^. These results revealed that cells were 400X to 2000X less viable in autophagy-dependent cancer cells than in non-malignant cells (Figure 1A-B). In addition, WX8 needs to be refreshed every two days to maintain nanomolar concentrations (Figure 2G), because inhibition by WX8 is reversible [8].

Remarkably, cellular levels of PIKFYVE protein varied only 3X with both the lowest and highest levels in the two most sensitive cell lines (Figure 1C, E). Therefore, WX8-sensitivity was not linked to PIKFYVE protein levels.

### WX8 sensitivity was not linked to differences in macroautophagy flux

Macroautophagy refers to degradation of cytoplasmic contents by engulfing them within autophagosomes which then fuse with lysosomes to form autolysosomes. The relative capacity of cells to carryout autophagy was quantified in nine different cell lines by comparing the extent to which WX8 induced accumulation of the autophagosome-associated protein LC3-II under optimal conditions [29]. LC3-II is a phosphatidylethanolamine conjugate of MAP1LC3/LC3-I that is unique to phagophore and autophagosomal membranes and an established indicator for autophagosome formation [30]. WX8 disrupts macroautophagy by preventing fusion between lysosomes and autophagosomes, which results in the accumulation of LC3-II protein and autophagosomes in the absence of autolysosomes [8]. Therefore, the relative levels of LC3-II accumulation reflect the relative accumulation of autophagosomes when macroautophagy is disrupted [31].

First, the concentration of WX8 that was required to accumulate the maximum amount of LC3-II protein in 4 h (the time required to form autolysosomes [32]) was determined for each cell line (Fig. S5A). Then this concentration of WX8 was used to determine the time required to produce the maximum amount of LC3-II protein in each cell line (Fig. S5B). Finally, the maximum levels of LC3-II were determined under optimal conditions for WX8 concentration and time for each cell line (Fig. S5C). These results revealed that the capacity for macroautophagy among different cell lines varied only 4X with both the lowest and highest levels in WX8-sensitive cell lines (Figure 1D, E). Therefore, WX8-sensitivity was not linked to differences in macroautophagy flux.

### WX8 sensitivity was not linked to the BRAF^V600E^ mutation

The BRAF^V600E^ oncoprotein is constitutively active in about 50% of all melanomas [33]. This mutation appears to promote autophagy-dependence in cultured cells [34] and autophagy-dependent melanomas in mice [35]. Moreover, *BRAF*^V600E^ pediatric brain tumors have been treated successfully with the autophagy inhibitor chloroquine [36]. However, the sensitivity of 10 different melanoma cell lines to WX8, as reflected by cellular ATP levels, ranged 667X. Remarkably, cells homozygous for *BRAF*^V600E^ were among the most sensitive as well as the most resistant to WX8 (Figure 1F, G). Therefore, WX8-sensitivity was not linked to the BRAF^V600E^ mutation.

### WX8 inhibited PIKFYVE and PIP4K2C in situ

To determine whether or not WX8-sensitivity of cells results from WX8 inhibiting the same proteins in cells (*in situ*) as it binds *in vitro*, melanoma A375 cells were cultured either in the presence of 0.05 µM or 1 µM WX8, or an equivalent volume of DMSO, the vehicle in which WX8 was dissolved. A375 cells are autophagy-dependent malignant cells that are highly sensitive to WX8 [8]. Concentrations up to 0.2 µM WX8 did not increase synthesis of LC3-II within 4 h, whereas concentrations ≥1 µM WX8 produce the maximum level of LC3-II within 4 h (Fig. S4E). WX8 activity was confirmed by the appearance of cytoplasmic vacuolization within 2 h in either low or high concentrations of WX8, and cell death began as early as 48 h in the presence of 1 µM WX8, as evidence by cells rounding up then detaching from the plate by 24 h to 48 h (Figures 2G; S4D), as previously reported [8]. Cell lysates were then treated for one hour with biotinylated acyl phosphates of ATP and ADP that react irreversibly with conserved lysine residues in the ATP-binding pocket of protein kinases, lipid kinases, and heat shock proteins [37,38]. The labeled peptides identified by mass spectrometry (MS).

Of the 312 peptides with active site modification, 92% were only 1.5-times more or less labeled in the presence of WX8 than in the presence of vehicle (Figure 2A). Only PIKFYVE was inhibited completely by either 0.05 µM or 1 µM WX8 (Figures 2B-D). PIP4K2C was the secondary target (26% with 0.05 µM increased to 52% with 1 µM), and CHUK/IKKA was the tertiary target [19% with 0.05 µM increased to 39% with 1 µM]. PIKFYVE and PIP4K2C were also the primary and secondary targets from 468 kinases previously profiled *in vitro* with 10 µM WX8 (Figure 2E) [8], and among the 24 kinases inhibited *in situ* from 18% to 97% (Fig. S6). The *in vitro* dissociation constants for WX8 from PIKFYVE, PIP4K2C and MTOR confirmed that only PIKFYVE and PIP4K2C preferentially bound WX8 (Figure 2F), and neither rapamycin inhibition of MTOR activity nor siRNA inhibition of *MTOR* gene expression induced either cytoplasmic vacuolization or inhibition of cell proliferation [8]. Therefore, WX8 specifically inhibited the same kinases *in situ* that it bound *in vitro.*

**Figure 3.**
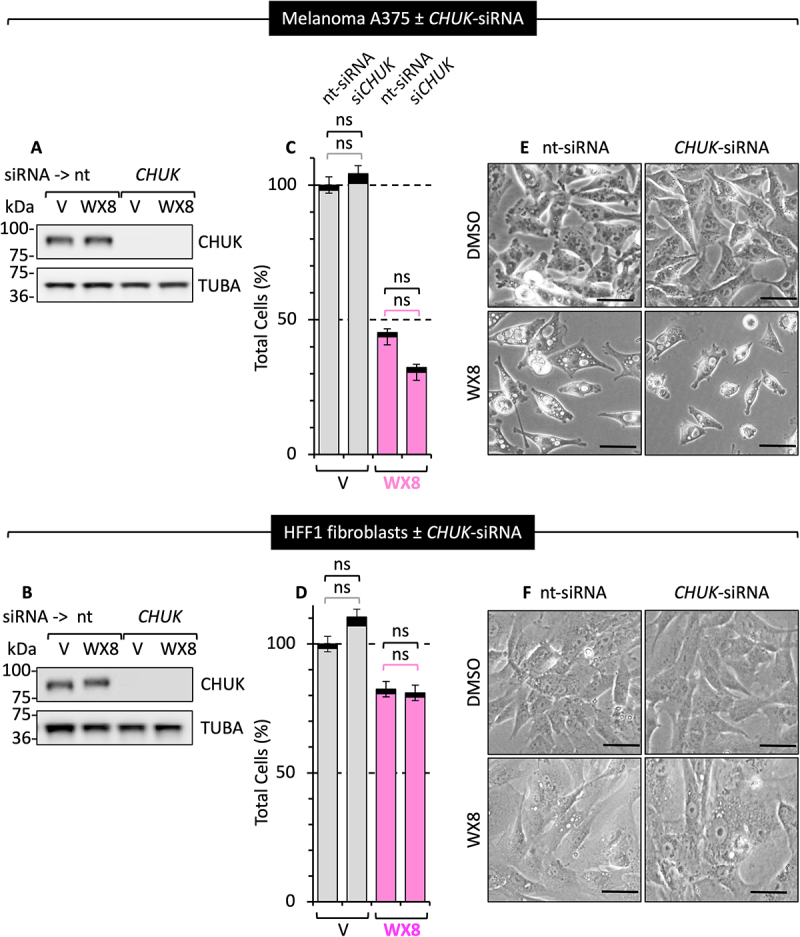
Inhibition of *CHUK/IKKA* gene expression did not enhance sensitivity to WX8. (A, B) A375 and HFF1 cells were transfected with either non-target (nt) or *CHUK*-siRNA and then cultured 72 hours in the presence of either vehicle or 0.05 µM WX8. Whole cell extracts were then immunoblotted to detect CHUK and TUBA proteins. (C, D) The percentage of live cells (trypan blue excluded) and dead cells (trypan blue stained) is indicated relative to vehicle-treated cells. (E, F) Phase contrast images of the cells in panel B (10X magnification). The numbers of live cells and dead cells were not affected by *CHUK*-siRNA followed by treatment with either vehicle or 0.05 µM WX8. Not significant (ns) is p > 0.05. Scale bar: 50 µm for A375 and 20 µm for HFF1 cells.

The tertiary target for WX8 *in situ* was the CHUK/IKKA serine kinase that regulates the NFKB/NF-kB signaling pathway and DNA damage response, thereby promoting cell proliferation, migration, and metastasis in human tumors [39]. Nevertheless, CHUK plays a minor role in maintaining the viability of autophagy-dependent cancer cells, because siRNA targeted against the *CHUK* gene (si*CHUK*) suppressed *CHUK* expression but did not affect proliferation of either A375 or HFF1 cells (Figure 3). Furthermore, si*CHUK* did not induce cytoplasmic vacuolization or affect cell proliferation, and selective inhibition of PIKFYVE activity by 0.05 µM WX8 together with si*CHUK* only marginally reduced A375 cell proliferation with no increase in cell death (Figure 3). None of the remaining proteins profiled *in vitro* were inhibited significantly *in situ* (Figs. S7; S8). Thus, the ability of WX8 to disrupt lysosome homeostasis and autophagy results from its ability to selectively inhibit the activities of the PIKFYVE and PIP4K2C kinases.

### Selective inhibition of PIKFYVE disrupted lysosome homeostasis

WX8 rapidly induces cytoplasmic vacuolization that results from inhibiting lysosome fission without inhibiting homotypic lysosome fusion [8]. This extent of cytoplasmic vacuolization depends on which PIKFYVE inhibitor is used, its concentration, and the length of time that cells are treated. With 0.05 µM WX8, the concentration at which only PIKFYVE was inhibited *in situ*, cytoplasmic vacuolization was evident within 2 hours but then disappeared between 72 and 96 hours (Figure 2G). Cytoplasmic vacuolization could be restored by changing to fresh medium with 0.05 µM WX8. However, with 1 µM WX8, the concentration at which both PIKFYVE and PIP4K2C were inhibited *in situ*, melanoma A375 cells underwent cell death, as evidenced by detaching from the plate and becoming permeable to trypan blue. In contrast, WX8-resistant HFF1 fibroblasts were also vacuolated by WX8, but they remained viable even with 1 µM WX8 (Figure 2H). Thus, disruption of lysosome homeostasis resulted solely from inhibition of PIKFYVE, as demonstrated previously with cells harboring a mutated *PIKFYVE* gene [9,10]. Furthermore, whereas low concentrations of PIKFYVE inhibitors can reduce cell proliferation, higher concentrations are required to induce cell death.

### Selective inhibition of PIKFYVE suppressed cell proliferation

**Figure 4.**
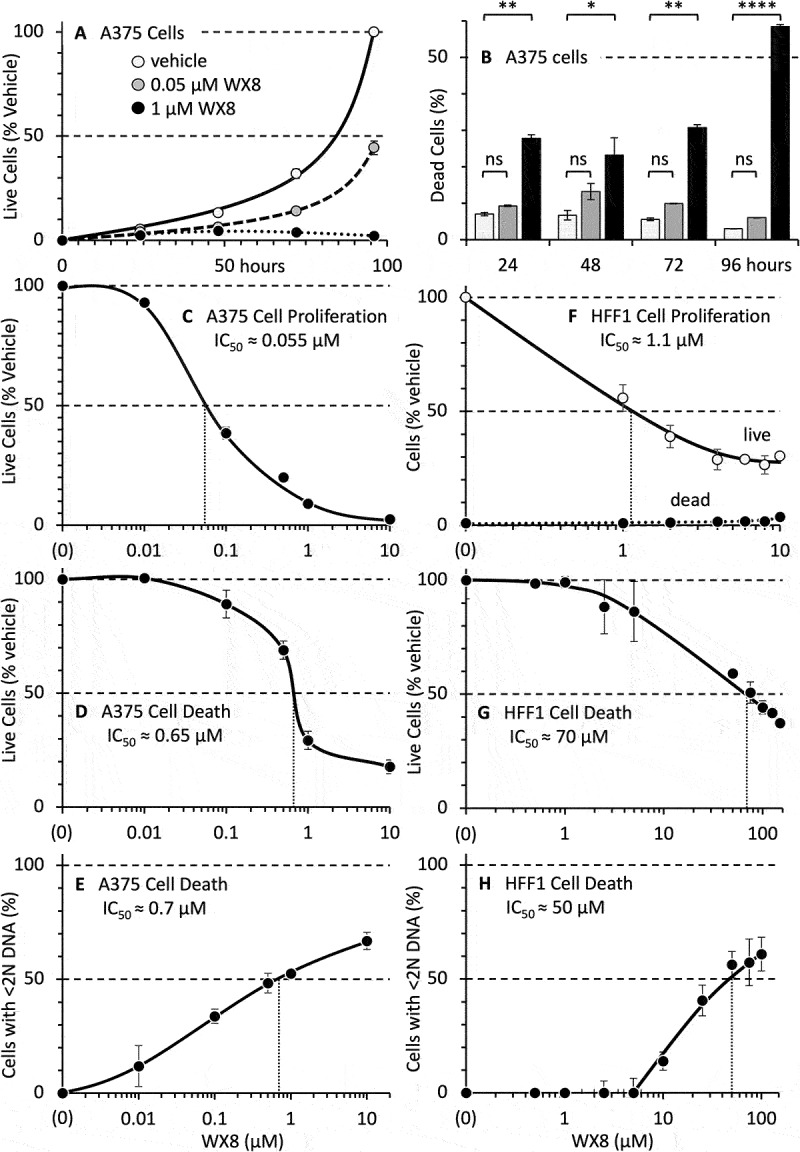
WX8 selectively inhibits proliferation and induces death in autophagy-dependent cells. (A) Autophagy-dependent melanoma A375 cells were most sensitive to WX8 when they were seeded at low density (Fig. S4A-C). Therefore, cells were seeded at 1428 cells/cm^2^ and then cultured for 24 h before adding either vehicle, 0.05 µM, or 1 µM WX8. The fraction of cells attached to the plate that excluded trypan blue was quantified as the fraction of live cells relative to the vehicle. (B) The fraction of unattached A375 cells stained with trypan blue was quantified as the fraction of dead cells relative to the vehicle. Light gray bars (vehicle), medium gray (0.05 µM WX8) and black (1 µM WX8). (C) The fraction of A375 cells attached to the plate that excluded trypan blue was quantified as the fraction of live cells relative to the vehicle. The IC_50_ for inhibition of cell proliferation was determined at 96 h. (D) The fraction of live A375 cells when stained with ANXA5 and propidium iodide after 96 h cultured with WX8. (E) The fraction of total cells (attached plus unattached cells) containing <2 N DNA (dead cells) was determined by fluorescence activated cell sorting (FACS) of cells cultured for 96 h with WX8. (F) HFF1 cells were treated as in panel A and then cultured for 96 h in the presence or absence of indicated concentrations of WX8. Open circles are live cells. Solid circles are dead cells. (G) The fraction of live HFF1 cells following staining with ANXA5 and propidium iodide after 96 h with WX8 (example Fig. S9C). (H) The fraction of dead HFF1 cells were defined as cells with <2 N DNA after culturing them for 96 h with WX8 (example Fig. S9E). To allow a logarithmic axis, 0 µM WX8 (vehicle) was plotted as 0.001 µM WX8 (C-E) or 0.1 µM WX8 (F-H). *, **, **** indicate statistical significance at p < 0.05, p < 0.01, p < 0.0001 level, respectively. Not significant (ns) is p > 0.05.

Treatment with 0.05 µM WX8 also inhibited A375 cell proliferation (Figure 4A) without inducing cell death (Figure 4B). The IC_50_ for inhibition of proliferation was 0.055 µM WX8 (Figure 4C). That was 12X less than the WX8 IC_50_ of 0.68 µM for accumulation of dead cells (Figure 4D, E). Thus, both PIKFYVE and PIP4K2C appeared to be required to induce cell death in WX8-sensitive cells.

Similar results were obtained with WX8-resistant cells, except that much higher WX8 concentrations were required. For example, the IC_50_ for WX8 induction of cell death in VeroE6 epithelial cells and HFF1 fibroblasts was 60 to 200-times greater than for inhibition of cell proliferation. The IC_50_ for cell proliferation was 0.30 µM for Vero6 and 1.1 µM for HFF1 (Figures 4F; S8B), whereas the IC_50_ for induction of cell death was about 60 µM for both Vero6 and HFF1 (Figures 4G, H; S8D, F).

### Inhibition of both PIKFYVE and PIP4K2C induced cell death selectively in WX8-sensitive cells

Induction of programmed cell death is characterized by the loss of plasma membrane lipid asymmetry, thereby exposing phosphatidylserine to ANXA5/annexin-V binding [40]. When the membrane become sufficiently permeabilized so that propidium iodide can enter and bind to DNA, cell death has been induced (example Fig. S9C). Live cells were also distinguished from dead cells by their ability to exclude trypan blue (Figure 4A–C). The IC_50_ for induction of programmed cell death was 70 µM WX8 for HFF1 (Figure 4G) and VeroE6 (Fig. S9D).

Cell death itself is marked by the accumulation of cells with less than 2N DNA content (<2N DNA) [41], as exemplified in figure S9E. This IC_50_ was 50 µM WX8 for HFF1 (Figure 4H) and 60 µM for VeroE6 (Fig. S9F). Thus, PIKFYVE-dependent cells were at least 100X more sensitive to induction of cell death than normal cells, suggesting that PIKFYVE-dependent cells required the PIK3-PIKFYVE-PIP4K2C pathway for synthesis of PtdIns(4,5)P_2_, whereas nonmalignant cells did not.

**Figure 5.**
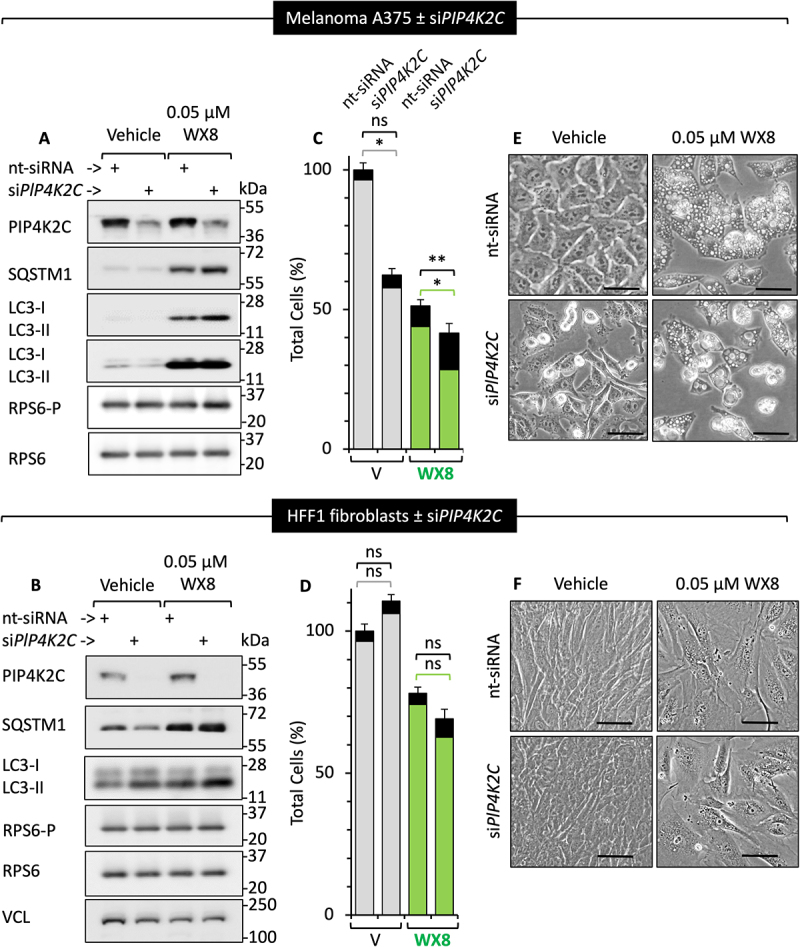
Induction of cell death by WX8 depended on PIP4K2C. (A, B) The effect of siRNA suppression of *PIP4K2C* (si*PIP4K2C*) on levels of SQSTM1, LC3-II (hallmarks of autophagic flux disruption), RPS6, and phosphorylated RPS6 protein (hallmarks of MTOR activity) relative to the effects with non-target siRNA (nt-siRNA). Assays were carried out in the presence of either 0.05 µM WX8 or DMSO either in A375 (sensitive) or HFF1 (resistant) cell lines. (C, D) The number of live cells (gray and green bars) and dead cells (black bars) from panels A and B are indicated relative to nt-siRNA. (E, F) Phase contrast images of attached cells cultured under the conditions indicated in panel A and B for either A375 or HFF1 cell lines. *, ** indicate statistically significance at the p < 0.05 and p < 0.01 level, respectively. Not significant (ns) is p > 0.05. Scale bar: 50 µm for A375 and 20 µm for HFF1 cells.

To determine the contribution of the PIP4K2C phosphoinositide kinase in induction of cell death, melanoma A375 cells were treated with siRNA against the *PIP4K2C* gene (si*PIP4K2C*) and then subsequently cultured in the presence of either vehicle or 0.05 µM WX8 to selectively inhibit PIKFYVE. Although si*PIP4K2C* significantly reduced PIP4K2C protein levels, neither LC3-II nor SQSTM1/p62 was induced in the absence of WX8 (Figure 5A). Only cytoplasmic vacuolation required the presence of WX8 (Figure 5E). Furthermore, neither si*PIP4K2C*, WX8 or a combination of si*PIP4K2C* and WX8 affected the cellular levels of either RPS6 or RPS6-P protein, whereas they did induce accumulation of both LC3-II and SQSTM1. Therefore, MTOR activity was not inhibited, as previously reported [8].

**Figure 6.**
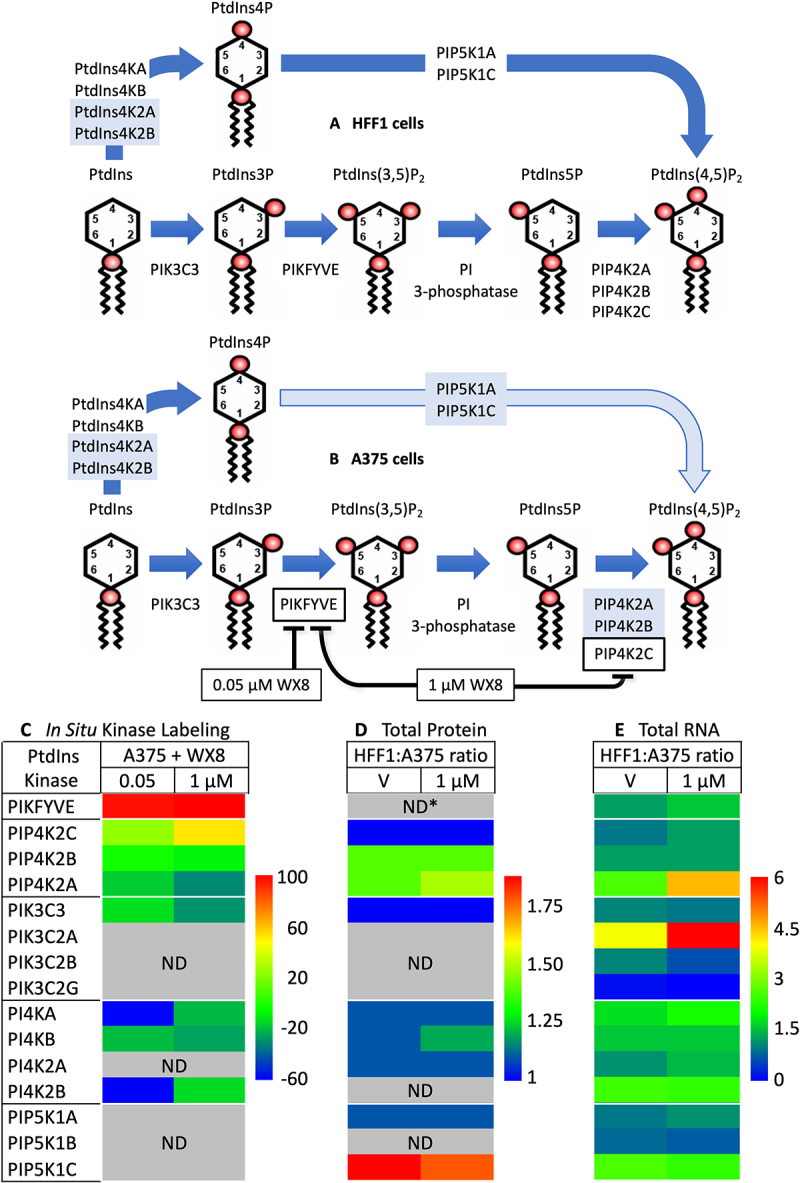
Phosphoinositide kinases involved in PtdIns(4,5)P_2_ synthesis and their sensitivity to WX8. (A) Two pathways produce PtdIns(4,5)P_2_ in WX8-resistant HFF1 cells. Phosphoinositides consist of a glycerol backbone esterified to two fatty acid chains and a phosphate (red ball) attached to inositol [adapted from [17]]. (B) The PIKFYVE-dependent pathway appears dominant in WX8-sensitive A375 cells. PtdIns-kinases that appear under-represented are shaded light blue. Names of PtdIns-kinases are in “Abbreviations” and figure S7. (C) Active PtdIns-kinases in A375 cells were labeled *in situ* after culturing for 4 h with either vehicle, 0.05 µM, or 1 µM WX8 under the same conditions used to detect *in situ* kinase inhibition by WX8 (Figure 2). Average percentage inhibition by WX8 from three independent *in situ* kinase assays is indicated as a heat map generated using HemI [74]. Gray boxes with “ND” indicate not detected (numerical data in Fig. S3B). (D) Relative abundance of PtdIns-kinases was detected by MS in HFF1 and A375 cells cultured as in Figure 2. Average relative abundance of PtdIns-kinases from three independent MS assays from both the cell lines are presented as a heat map (numerical data in Fig. S3C). (ND*) PIKFYVE-VAC14-FIG4 is the active form of the PIKFYVE phosphoinositide kinase, but only VAC14 and FIG4 were identified. (E) Relative number of specific gene transcripts per million total transcripts were determined for HFF1 (n = 3) and A375 (n = 4) cells cultured either in presence of vehicle or 1 µM WX8 as in Figure 2. Ratios of HFF to A375 are indicated as a heat map (numerical data in Fig. S3C).

SQSTM1 recruits aggregate-prone proteins to autophagosomes [29]. Thus, PIP4K2C and PIKFYVE have distinctly different roles. However, suppression of PIP4K2C protein levels by si*PIP4K2C* together with inhibition of PIKFYVE by WX8 further reduced cell proliferation with a concomitant increase in cell death (Figure 5C). Thus, the functions of PIKFYVE and PIP4K2C are synergistic, consistent with their roles in PtdIns(4,5)P_2_ synthesis (Figure 6).

In contrast with A375 cells, neither proliferation nor death of HFF1 cells was affected by the loss of PIP4K2C protein (Figure 5B, D), and the effects of WX8 on HFF1 cell proliferation, cell death, and cytoplasmic vacuolization were unaffected by the loss of PIP4K2C protein (Figure 5B-F). Thus, PIKFYVE-dependent cells required the PIK3-PIKFYVE-PIP4K2C pathway for synthesis of PtdIns(4,5)P_2_, whereas nonmalignant cells did not.

### The effects of WX8 were not linked to regulation of PtdIns-kinase gene expression

Given that autophagy-dependent cells required PIKFYVE and PIP4K2C activities under conditions wherein non-malignant differentiated cells did not, WX8 sensitivity appeared to result from differences in their ability to synthesize PtdIns(4,5)P_2_. The human genome encodes 19 PtdIns-kinases engaged in eight different metabolic events [42], but only 12 PtdIns-kinases are involved in converting PtdIns into PtdIns(4,5)P_2_ via the two known pathways [13,17,18] (Figure 6A).

To determine which PtdIns-kinase genes were expressed in WX8-sensitive and resistant cells, RNA sequences were profiled in both A375 and HFF1 cells, cultured with either vehicle or 1 µM WX8. All 12 of the PtdIns-kinases involved in converting PtdIns into PtdIns(4,5)P_2_ were among the 37,936 RNAs identified in both A375 and HFF1 cells, and the ratios of PtdIns-kinase RNAs in HFF1 cells relative to A375 cells were calculated from the number of transcripts of a single gene per million total transcripts (heat map Figure 6E; numerical values Fig. S3C). The change in RNA levels was also calculated as the log2 of the fold change (Fig. S3D,E).

None of the RNA levels from the 12 PtdIns-kinase genes that facilitate PtdIns(4,5)P_2_ synthesis were upregulated in A375 cells by WX8. Neither were they downregulated (data not shown). In HFF1 cells, only *PIP4K2A* RNA levels increased significantly in the presence of WX8 (1.8X in total RNA; 1.17 in log_2_FC). Therefore, the effects of WX8 on sensitive and resistant cells were not linked to the regulation of PtdIns-kinase gene expression.

### The effects of WX8 were linked to differences in PtdIns-kinase protein levels

To determine which PtdIns-kinase proteins were present in WX8-sensitive and resistant cells, PtdIns-kinases identified in WX8-treated A375 cells using a biotin-ATP probe were compared with PtdIns-kinases identified by MS analysis of total cell extracts from both A375 and HFF1 cells. Only 11 of the kinases identified in the 246 kinases in dendrogram of melanoma A375 cells labeled with Biotin-ATP were PtdIns-kinases (Fig. S7). Of these, only eight were involved with PtdIns(4,5)P_2_ synthesis (heat maps Figure 6C, numerical data Fig. S3B). These results suggested that A375 cells are deficient in the three PIP5K1 isozymes required to convert PtdIns4P into PtdIns(4,5)P_2_.

To compare the PtdIns-kinases in A375 cells with the PtdIns-kinases in HFF1 cells, both cell lines were cultured for 16 to 18 hours in the presence of either vehicle or 1 µM WX8. Of the 6,736 proteins identified by MS of total cell extracts, 12 were PtdIns-kinases (Figs. S3C; S7). Of these, nine were involved with PtdIns(4,5)P_2_ synthesis, and seven were not detected (Figure 6D; Fig. S3C). Remarkably, PIKFYVE protein was not detected. However, since PIKFYVE inhibitors induce cytoplasmic vacuolization in resistant cells as well as sensitive cells, PIKFYVE protein must be present, but in low abundance. The functional form of PIKFYVE is a complex with VAC14 and FIG4 [43], both of which were detected by MS analysis of whole cell extracts (Fig. S3C). PI4K2B also appears to be a low abundance protein because it appears in low abundance in HFF1 cells, but neither PIP5K1B protein nor *PIP5K1B* RNA was detected in A375 cells.

Taken together with the *in situ* kinase labeling results, PtdIns(4,5)P_2_ synthesis in both cell lines appeared to occur in the absence of PIP5K1B and a deficiency in PI4K2A and PI4K2B. But the distinction between WX8-sensitive A375 cells and WX8-resistant HFF1 cells appeared to result from an A375 deficiency in the three PIP5K1 isozymes that convert PtdIns4P into PtdIns(4,5)P_2_ and in the PIP4K2A and B isozymes that convert PtdIns5P into PtdIns(4,5)P_2_. These deficiencies would allow inhibition of PIKFYVE and PIP4K2C to selectively prevent PtdIns(4,5)P_2_ synthesis in A375 cells (Figure 6B) but not in HFF1 cells (Figures 6A).

Reducing the PtdIns(4,5)P_2_ pool requires more than simply inhibiting PIKFYVE activity. Suppression of *PIKFYVE* gene expression in mouse embryonic fibroblasts [17] and apilimod inhibition of PIKFYVE activity in human HeLa cells [44] suppressed the PtdIns(3,5)P_2_ pool and increased the PtdIns3P pool, but did not reduce the PtdIns(4,5)P_2_ pool. Similarly, PIP4K2C specific inhibitors increase the levels of upstream phosphoinositides PtdIns(3,5)P_2_, PtdIns5P, and PtdIns3P in HEK293T cells without affecting the levels of PtdIns, PtdIns4P or PtdIns(4,5)P_2_ [45]. These results are consistent with two active pathways for PtdIns(4,5)P_2_ synthesis, one from PtdIns4P and one from PtdIns3P (Figure 6A). Thus, selective termination of WX8-sensitive cells is a consequence of the fact that 1 µM WX8 inhibits both PIKFYVE and PIP4K2C (Figure 2), and the fact that WX8-sensitive cells are deficient in PIP5K1C, the enzyme required to convert PtdIns4P into PtdIns(4,5)P_2_ (Figure 6).

### PIKFYVE-dependent cells were deficient in PIP5K1C

**Figure 7.**
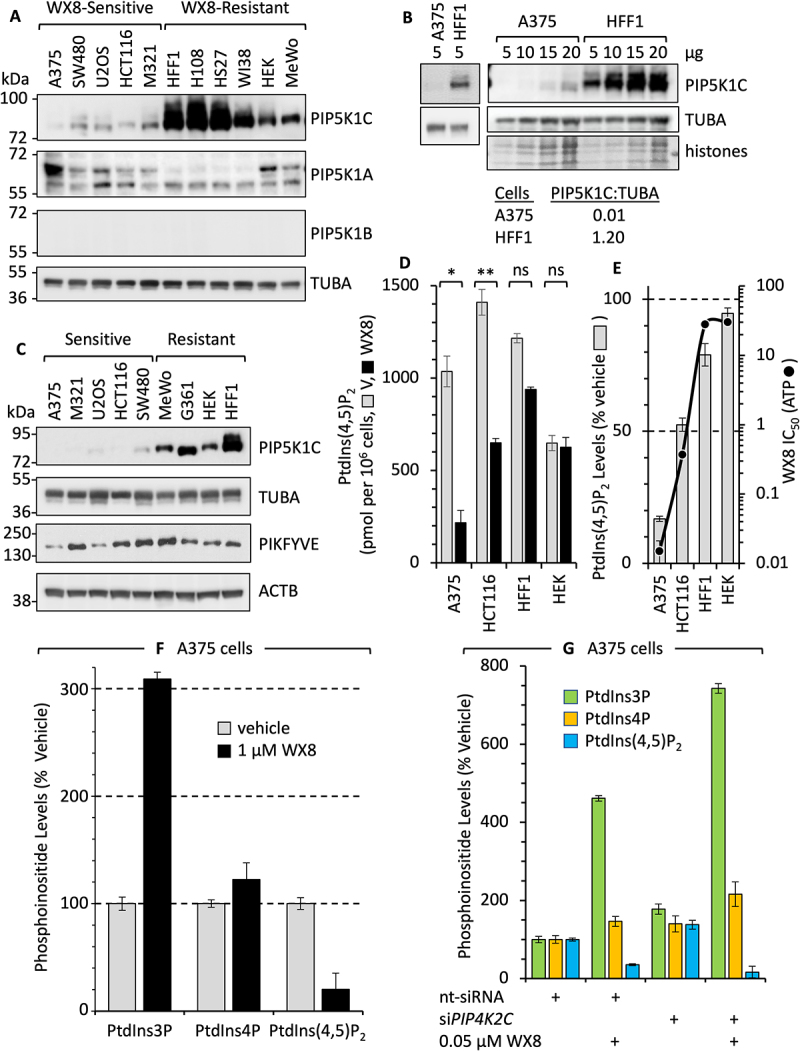
PIP5K1C protein and phosphoinositide biosynthesis in WX8-sensitive and insensitive cells. (A) Immunoblots of the three PIP5K1 isozymes in WX8-sensitive and resistant human cell lines. Proteins were identified by a specific antibody and by their molecular weight relative to protein standards. PIP5K1B was not detected in any of the samples. (B) The indicated amounts of total cell protein from A375 and HFF1 cells were immunoblotted for PIP5K1C. Side by side comparison of 5 µg samples from A375 and HFF1 cells exposed for less time were quantified by densitometry. Ratios of PIP5K1C:TUBA are indicated. (C) PIP5K1C immunoblot of the same cell lines used to quantify PIKFYVE protein in Figure 1C. About 2X less PIP5K1C sample was applied to this gel as to gel in panel A. (D) The indicated cell lines were cultured for 24 h with either vehicle or 1 µM WX8. Total PtdIns(4,5)P_2_ (pmoles/10^6^ cells) was quantified by ELISA assays. (E) The percentage decrease of PtdIns(4,5)P_2_ in different cells after WX8 treatment compared with cell viability as quantified by ATP loss. (F) The percentage change in the levels of PtdIns3P, PtdIns4P and PtdIns(4,5)P_2_ in A375 cells cultured with 1 µM WX8 for 24 hours. (G) The percentage change in the levels of PtdIns3P, PtdIns4P and PtdIns(4,5)P_2_ in A375 cells cultured for 72 h with no-target siRNA, or siRNA against *PIP4K2C* (si*PIP4K2C*) either with or without 0.05 µM WX8. Phosphoinositides were quantified by ELISA assays.

To determine whether or not cellular levels of PIP5K1C distinguished WX8-sensitive cells from WX8-resistant cells, whole cell extracts of 11 different cell lines were subjected to immunoblotting for PIP5K1A, B, and C (Figure 7A). The results confirmed that PIP5K1B protein was not expressed in either sensitive or resistant cells, and that PIP5K1C protein was deficient in WX8-sensitive cells relative to resistant cells. Comparison of PIP5K1C levels at different gel loading concentrations revealed that PIP5K1C protein was at least 120X greater in HFF1 cells than in A375 cells (Figure 7B). Comparison between PIP5K1C and PIKFYVE immunoblots confirmed that the distinction between WX8-sensitive and resistant cells was linked to PIP5K1C levels and not to PIKFYVE levels (Figure 7C).

These differences were reflected in the effect of WX8 on cellular levels of PtdIns(4,5)P_2_ and PtdIns3P. In the absence of WX8, cellular levels of PtdIns(4,5)P_2_ varied by only 2X (Figure 7D, gray bars). In the presence of 1 µM WX8, the level of PtdIns(4,5)P_2_ in A375 cells was reduced by 83% and HCT116 by 50% whereas the level of PtdIns(4,5)P_2_ in HFF1 was reduced by 20% and HEK by only 3% (Figure 7D, black bars). Moreover, inhibition of PtdIns(4,5)P_2_ synthesis by WX8 was inversely proportional to the IC_50_ for WX8 suppression of ATP levels, revealing that sensitivity of PtdIns(4,5)P_2_ synthesis to WX8 is linked directly to sensitivity of cell viability to WX8 (Figure 7E). WX8 also induced a 3X increase in PtdIns3P, the substrate for PIKFYVE, without affecting PtdIns4P levels (Figure 7F). Thus, WX8 appears to inhibit cell viability by simultaneously suppressing PtdIns(4,5)P_2_ levels and elevating PtdIns3P levels.

To distinguish the influence of each phosphoinositide kinase on the levels of PtdIns3P, PtdIns4P and PtdIns(4,5)P_2_, A375 cells were treated with either 0.05 µM WX8 to inhibit PIKFYVE activity, or si*PIP4K2C* to suppress *PIP4K2C* expression, or a combination of both. With 1 µM WX8, both PIKFYVE and PIP4K2C activities were inhibited, but with 0.05 µM WX8, only PIKFYVE was inhibited. Concurrent inhibition of both PIKFYVE and PIP4K2C significantly increased the cellular level of PtdIns3P by ~1.6X, and it decreased the level PtdIns(4,5)P_2_ by ~2X, but it had no significant change in PtdIns4P levels (Figure 7G). These results were consistent with the effects of 1 µM WX8 (Figure 7F), which inhibited 97% of PIKFYVE and 52% of PIP4K2C (Figures 2; S6).

Thus, the extent to which WX8 disrupts lysosome homeostasis and macroautophagy through changes in phosphoinositide levels is a consequence of both the lack of PIP5K1C activity and the extent to which WX8 inhibits the activities of both the PIKFYVE and PIP4K2C kinase.

### PIP5K1C was essential for WX8-resistance

The essential role of PIP5K1C in preventing WX8-induced cell death was confirmed by siRNA suppression of *PIP5K1C* gene expression (si*PIP5K1C*), chemical inhibition of PIP5K1C kinase activity, and ablation of the *PIP5K1C* gene in WX8-resistant HFF1 cells. In each case, loss of PIP5K1C activity converted WX8-resistant cells into WX8-sensitive cells.

**Figure 8.**
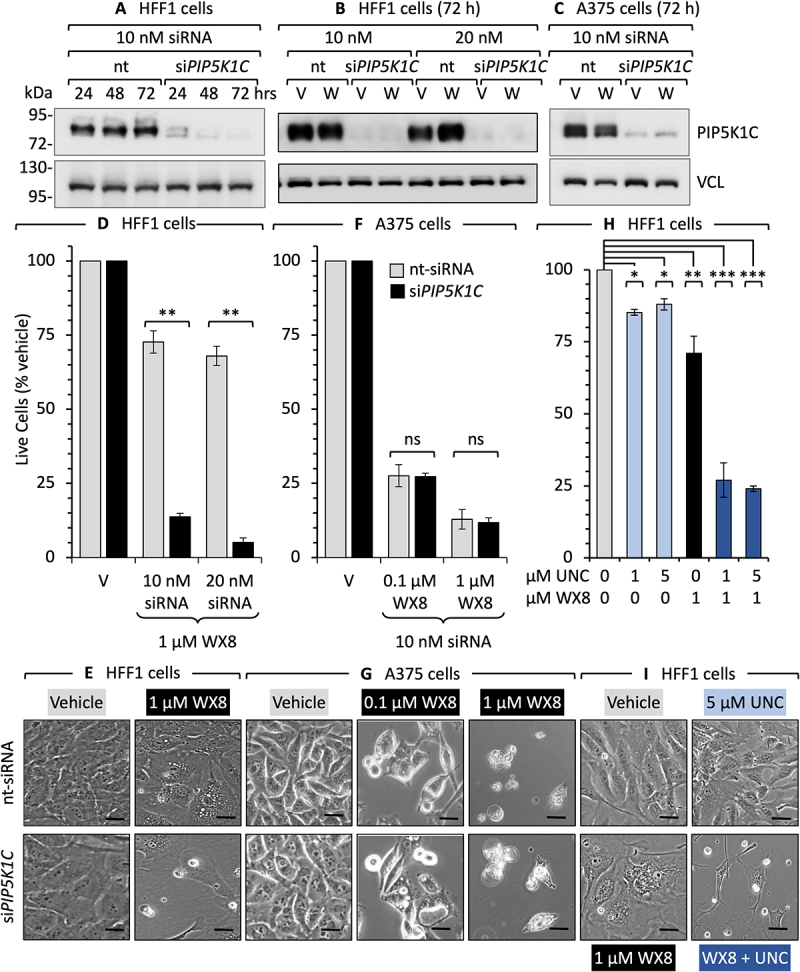
siRNA suppression of *PIP5K1C* in WX8-resistant cells transformed them into WX8-sensitive cells. (A) PIP5K1C was quantified in HFF1 cells by immunoblotting at different times after addition of either non-targeted siRNA (nt) or siRNA targeted against the *PIP5K1C* gene. (B) Immunoblotting PIP5K1C protein 72 h after addition of increasing amounts of siRNA targeted against *PIP5K1C* gene expression in HFF1 cells cultured with either vehicle (V) or 1 µM WX8 (W). (C) Immunoblotting of PIP5K1C protein 72 h after addition of siRNA (10 nM) against *PIP5K1C* gene in A375 cells cultured with either vehicle (V) or 0.1 µM WX8. More protein and longer exposure times were used to detect the basal level of PIP5K1C in A375 cells by immunoblotting. (D) Fraction of live HFF1 cells relative to vehicle in panel B. (E) Phase contrast images of the cells in panel D (scale bar: 20 µm). (F) Fraction of live A375 cells relative to vehicle in panel C. (G) Phase contrast images of the cells in panel F. ** indicates statistical significance at *P *< 0.001. Not significant (ns) is p > 0.05. (scale bar: 50 µm). (H) Total live HFF1 cells (trypan blue excluded) were quantified after 72 h of culture in the presence of the indicated combinations of vehicle (V), WX8 (1 µM) and UNC3230 (5 µM). UNC3230 is a specific inhibitor of PIP5K1C [46]. *, **, *** indicate statistical significance at *p *< 0.05, *p* < 0.01, *p* < 0.001 level, respectively. Not significant (ns) is p > 0.05. (I) Phase contrast images of the HFF1 cells in panel H treated as indicated. All microscopy images are 10X magnification (scale bar: 20 µm).

The level of PIP5K1C protein in HFF1 cells was suppressed completely with si*PIP5K1C* (Figure 8A, B). Under these conditions, neither HFF1 cell proliferation nor cell death was affected (Figure 8D). However, addition of 1 µM WX8 reduced live cells by 93% of control. si*PIP5K1C* neither induced cytoplasmic vacuolization nor prevented cytoplasmic vacuolization induced by WX8 (Figure 8E). Thus, PIP5K1C was essential for WX8-resistance, but not for lysosome homeostasis.

As with HFF1 cells, siRNA suppression of PIP5K1C protein in WX8-sensitive A375 cells (Figure 8C) did not affect the ability of WX8 to induce cytoplasmic vacuolization (Figure 8G). However, in contrast with HFF1 cells, WX8 inhibited cell proliferation of A375 cells at low concentrations and terminated cell viability at higher concentrations (Figure 8F,G).

To distinguish between loss of PIP5K1C protein and loss of PIP5K1C enzyme activity, HFF1 cells were treated with UNC3230, a specific inhibitor of PIP5K1C kinase activity [46]. UNC3230 reduced proliferation of HFF1 cells by 10–15%, but it increased their sensitivity to WX8 by 76% (Figure 8H). Consistent with its target specificity, UNC3230 neither induced cytoplasmic vacuolization nor prevented WX8 from inducing vacuolization (Figure 8I). Consequently, HFF1 cells cultured with both UNC3230 and WX8 died.

**Figure 9.**
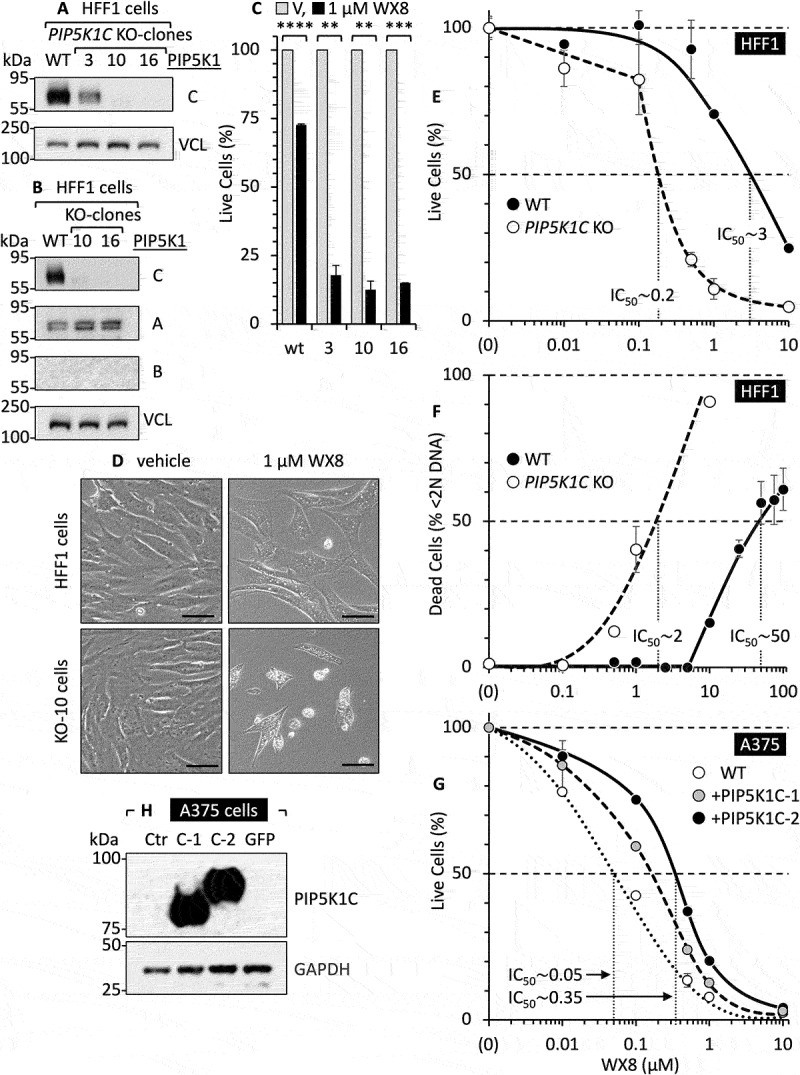
PIP5K1C was essential to prevent WX8-induced cell death. (A) Immunoblot of PIP5K1C protein in three different clones derived from HFF1 cells in which *PIP5K1C* was ablated using Crisper-Cas9 technology. (B) Immunoblot of PIP5K1 isozymes in *PIP5K1C* knockout cell lines. (C) Percentage of live cells in 1 µM WX8-treated cells compared to vehicle (V) in both in WT and *PIP5K1C* “knockout” (KO) clones. **, ***, **** indicate statistical significance at P < 0.01, 0.001, 0.0001, respectively. (D) Phase-contrast images showing WT and *PIP5K1C* KO cells grown in the presence of either vehicle or 1 µM WX8 (scale bar: 20 µm). (E) Percentage of live cells compared to vehicle in both WT and *PIP5K1C* KO cells. (F) Percentage of dead cells compared to vehicle in both WT and *PIP5K1C* KO cells. (G) PIP5K1C overexpression in A375 cells. PIP5K1C-1 and PIP5K1C-2 represent isoform 1 and 2, respectively. (H) Western-immunoblot showing the overexpression of PIP5K1C isoforms in A375 cells.

To confirm that PIP5K1C was essential for WX8-resistance, the *PIP5K1C* gene was ablated in HFF1 cells, and HFF1 (*PIP5K1C^−/−^*) clones were isolated. PIP5K1B was not detected either in HFF1 cells or any of the *PIP5K1C^−/−^* clones, whereas PIP5K1A was expressed in HFF1 cells and all of the *PIP5K1C^−/−^* clones. Three clones were selected for further analysis (Figure 9A, B). Clone 3 expressed reduced levels of PIP5K1C (suggesting heterozygosity), whereas PIP5K1C was not detected in clones 10 and 16. Remarkably, all three clones were 8X more sensitive to WX8 than wild-type HFF1 cells with 85% to 91% reduction in live cells (Figure 9C). PIP5K1C^−/−^ cells remained sensitive to WX8 induction of cytoplasmic vacuolation and inhibition of cell proliferation (Figure 9D). However, the IC_50_ values for WX8 inhibition of cell proliferation (Figure 9E) and cell death (Figure 9F) of HFF1(*PIP5K1C^−/−^*) cells were about 20X less than for HFF1 wild-type cells. Taken together, these results demonstrated that a PIP5K1C deficiency is what distinguishes WX8-sensitive, PIKFYVE-dependent, cancer cells from WX8-resistant non-malignant cells.

To confirm the role of PIP5K1C in WX8 mediated sensitivity, PIP5K1C was transiently expressed in melanoma A375 cells. However, several isoforms of PIP5K1C have been reported and associated with specific functions [47]. Two of them (PIP5K1C-1 and PIP5K1C-2) were detected in HFF1 cells using isoform specific PCR-primers. Therefore, *PIP5K1C-1* and *PIP5K1C-2* isoforms were each cloned into a DNA expression vector and transfected into A375 cells where they over-expressed their PIP5K1C isoform protein relative to untransfected cells and to cells transfected with the same vector expressing green fluorescence protein (Figure 9H). Despite the fact that overexpressed PtdIns-kinases lose their normal targeting to different cellular compartments [48,49], the IC_50_ for WX8 inhibition of cell proliferation was seven-fold greater in cells expressing the PIP5K1C-1 isoform than in control cells (Figure 9G).

## Discussion

Autophagy-dependent cancer cells are much more sensitive than non-malignant cells to WX8 and related PIKFYVE inhibitors when quantified by a loss of cellular ATP (~2000X), cell proliferation (~50X), cell permeability (~65X) or genomic DNA (~75X) [8–10] (Figure 1). In the present study, six hypotheses were investigated initially to determine what could account for these differences, but none of them could. First, WX8 neither upregulated nor downregulated *PIKFYVE* RNA levels in either WX8-sensitive or resistant cells. Second, PIKFYVE protein levels varied only 3X among 11 different cell lines with no correlation to WX8-sensitivity. In fact, one of the most sensitive cell lines contained the most PIKFYVE protein. Third, autophagic flux varied only 4X, and it was sometimes greater in sensitive cells than in resistant cells. Fourth, WX8-sensitivity was not linked to the *BRAF^V600E^* mutation found in >50% of melanoma and other cancers [50], because cells homozygous for *BRAF^V600E^* were among the most resistant to WX8 as well as the most sensitive. Similar results have been reported for oncogenic *KRAS* mutations [51]. Fifth, WX8 neither upregulated nor downregulated PtdIns-kinase RNA levels in either WX8-sensitive or resistant cells. Finally, WX8-sensitivity did not result from ambiguous inhibitor specificity, because the two proteins selectively bound by WX8 and its chemical analogs *in vitro* (PIKFYVE and PIP4K2C, Fig. S2) were also selectively inhibited by WX8 *in situ* (Figure 2). Neither siRNA suppression of *MTOR* expression (the tertiary target *in vitro*) nor *CHUK* (the tertiary target *in situ*) expression induced cytoplasmic vacuolization or inhibited cell proliferation, and none of the weaker protein targets *in vitro* corresponded to weaker protein targets *in situ* (Figures 2, S6).

The ability of WX8 to selectively inhibit PIKFYVE at low concentrations and both PIKFYVE and PIP4K2C at higher concentrations revealed that the distinction between PIKFYVE-dependent (autophagy-dependent) cancer cells and nonmalignant cells resulted from a deficiency in PIP5K1C that prevented nonmalignant cells from producing PtdIns(4,5)P_2_ in the absence of PIKFYVE. This discovery suggests that clinical applications of PIKFYVE inhibitors could be identified in cancer cells by a low abundance of PIP5K1C relative to non-malignant cells.

### Phosphoinositide kinases

Analyses of the phosphoinositide kinases in WX8-sensitive and resistant cells confirmed the existence of two pathways for PtdIns(4,5)P_2_ synthesis (Figure 6), A phosphoinositide believed to be required on endo-lysosomal membranes in order to maintain lysosome homeostasis, endosomal trafficking, and autophagic flux [19–21]. Of the 19 PtdIns-kinases in the human genome (Fig. S7), 11 were detected by *in situ* labeling of their active site, 13 by MS of whole cell extracts and 18 by RNA sequence of whole cell extracts (Fig. S3D, E). Of these, only 11 were involved in PtdIns(4,5)P_2_ synthesis (Figure 6A). Of these, only PIK3C3, PIKFYVE, and PIP4K2C are required for efficient conversion of PtdIns -> PtdIns3P -> PtdIns5P -> PtdIns(4,5)P_2_; PIP4K2A and B appear to play supporting roles in PIKFYVE-dependent cells (Figure 6B). PI4KA and B, and to a lesser extent PI4K2A and B, convert PtdIns -> PtdIns4P. Only PIP5K1A and C convert PtdIns4P -> PtdIns(4,5)P_2_. PIP5K1C is the primary catalyst in nonmalignant cells, but deficient in PIKFYVE-dependent cells. Thus, autophagy-dependent cancer cells require both PIKFYVE and PIP4K2C to produce PtdIns3P, PtdIns5P, and PtdIns(4,5)P_2_, because they are deficient in PIP5K1C. Consequently, WX8 selectively terminates PIKFYVE-dependent cancer cells by virtue of its ability to selectively inhibit both PIKFYVE and PIP4K2C.

WX8-sensitive cells contained ~120X less PIP5K1C protein than resistant cells (Figure 7A-C), and neither PIP5K1A nor PIP5K1B appear to contribute to WX8-resistance. Conversion of PtdIns4P into PtdIns(4,5)P_2_ requires at least one of the three PIP5K1 isozymes. PIP5K1B protein was not detected in any of cell lines tested by labeling ATP binding sites (Figure 6C), by MS analysis of total cellular proteins (Figure 6D), or by immunoblotting with PIP5K1B antiserum (Figure 7A). In contrast, PIP5K1A protein was detected in all of cell lines tested, but it did not appear to contribute to WX8-resistance, because no link was detected between PIP5K1A protein levels and WX8 sensitivity. PIP5K1A protein was detected by MS in A375 cell extracts but not by in situ kinase labeling in A375 cells (Figure 6C, D). Moreover, A375 cells were more sensitive than HCT116 cells to WX8 despite the fact that A375 contained significantly more PIP5K1A protein (Figure 7A). In fact, WX8 reduced PtdIns(4,5)P_2_ levels more effectively in A375 cells than in HCT116 cells (Figure 7D). Thus, the primary, perhaps exclusive, link between WX8-sensitive and resistant cells is the level of PIP5K1C activity.

This conclusion was confirmed by the fact that loss of PIP5K1C activity in WX8-resistant cells converted them into WX8-sensitive cells, and ectopic expression of PIP5K1C protein in WX8-sensitive cells converted then into WX8-reistant cells (Figure 9G). The PIP5K1C kinase inhibitor UNC3230 had little effect on HFF1 cells unless accompanied by WX8, in which case cell death was induced. siRNA against *PIP5K1C* also increased WX8 sensitivity in HFF1 cells >90%, but it had no effect on WX8 sensitivity in A375 cells. These results were confirmed by gene ablation. Inhibition of cell proliferation in HFF1(*PIP5K1C^−/−^*) cells was 15X more sensitive to WX8 and induction of cell death was 20X more sensitive. Ablation of *PIP5K1C* in human HEK293 cells also results in accumulation of autolysosomes, thereby disrupting autophagic flux [52,53]. Conversely, transient expression of either isoform of *PIP5K1C* in WX8-sensitive A375 cells increased their resistance to WX8.

### Phosphoinositide pools

The fact that both PIKFYVE and PIP4K2C are required for efficient PtdIns(4,5)P_2_ synthesis in PIKFYVE dependent cells was confirmed by suppressing expression of *PIP4K2C* with siRNA while selectively inhibiting PIKFYVE with 0.05 µM WX8 (Figure 7G). si*PIP4K2C* alone did not significantly affect the levels of PtdIns3P, PtdIns4P or PtdIns(4,5)P_2_. However, in combination with WX8, the level of PtdIns3P increased and the level of PtdIns(4,5)P_2_ decreased. Minor increases in PtdIns4P presumably reflect the cell’s effort to overcome a catastrophic loss of PtdIns(4,5)P_2_, suggesting that PtdIns(4,5)P_2_ is essential for macroautophagy.

Inhibition of PIKFYVE should also reduce cellular levels of PtdIns(3,5)P_2_ and PtdIns5P, because PtdIns(3,5)P_2_ is synthesized by PIKFYVE, and PtdIns5P is produced either from PtdIns(3,5)P_2_ by a 3’-phosphatase or synthesized directly by PIKFYVE [17,18]. Inhibition of both PIKFYVE and PIP4K2C should also reduce cellular levels of PtdIns(3,4,5)P_3_, which is synthesized from PtdIns(4,5)P_2_ by PIK3C [42]. Whether or not these changes in phosphoinositides are important to cell viability is the subject of future studies.

The effects of WX8 on cell viability appear to be a consequence of its effects on phosphoinositides that are essential for lysosome homeostasis and macroautophagy. In the presence of WX8, PtdIns(4,5)P_2_ pools were reduced in proportion to the cell’s metabolic sensitivity to WX8 (Figure 7E), suggesting that inhibition of cell proliferation and lysosome homeostasis in WX8-sensitive cells results from inhibition of PtdIns(4,5)P_2_ synthesis. PIKFYVE synthesizes PtdIns(3,5)P_2_ and PtdIns5P [18], two low abundance phosphoinositides that play critical roles in lysosome homeostasis, membrane trafficking, autophagy, and transcription [43]. PtdIns(3,5)P_2_, is also a precursor in PtdIns(4,5)P_2_ synthesis, a phosphoinositide required for fusion of lysosomes with autophagosomes to produce autolysosomes [19–21], as well as for ion channel regulation, intracellular trafficking, vesicular transport, actin and adhesion dynamics, and the DNA damage response [54–57]. Thus, PtdIns(4,5)P_2_ appears to play a critical role in maintaining cell growth and proliferation.

### Cell proliferation and cell death

Selective inhibition of PIKFYVE activity arrests cell proliferation without inducing cell death. *PIKFYVE* is a unique, haploid sufficient, gene whose ablation results in cell cycle arrest [13]. *PIKFYVE* nullizygous mouse embryos survive until the blastocyst stage, presumably from maternally inherited PIKFYVE protein, but embryonic fibroblasts derived from Cre-induced *PIKFYVE* ablation develop cytoplasmic vacuolization and arrest cell division [58]. Similarly, deletion of the *Fab1/PIKFYVE* gene in yeast impairs nuclear division, resulting in aneuploid and binucleate cells [59].

Selective inhibition of PIKFYVE activity by culturing WX8-sensitive melanoma A375 cells in 0.05 µM WX8 arrested cell proliferation without inducing cell death (Figure 4). Cell death was induced in A375 cells only when both PIKFYVE and PIP4K2C were inhibited with ≥1 µM WX8, or when cells were treated with both 0.05 µM WX8 to selectively inhibit PIKFYVE and siRNA to selectively inhibit *PIP4K2C* gene expression. Thus, ATP loss and inhibition of A375 cell proliferation occurred with an IC_50_ of 0.05 µM WX8, whereas plasma membrane permeability and DNA loss occurred with an IC_50_ of 0.68 µM WX8.

Induction of cell death by PIKFYVE inhibitors, as quantified by ANXA5 binding, plasma membrane permeability, and cellular DNA loss, not only requires higher concentrations of PIKFYVE inhibitors, but it begins 10 h to 15 h after cytoplasmic vacuolization occurs [11]. Thus, the onset of cell death is coincident with disruption of “macroautophagy”, an event recognized by a concomitant accumulation of LC3-II and SQSTM1 [9–11].

Although HFF1 cells are resistant to WX8 relative to A375 cells, WX8 inhibits HFF1 proliferation with an IC_50_ of 1 µM WX8, which is 50-times greater than for A375 cells. Similarly, the IC_50_ for HFF1 cell death, as quantified by plasma membrane permeability, ANXA5 binding, and DNA loss is at least 50-times greater than A375. Thus, induction of cell proliferation occurs by inhibition of PIKFYVE, whereas induction of cell death requires additional events.

### Secondary targets of PIKFYVE inhibitors

PIKFYVE is the primary target of the group of established PIKFYVE inhibitors for which WX8 is the prototype, and PIP4K2C is the secondary target (Fig. S2) [8]. All three PIP4K2 isozymes were detected in both A375 and HFF1 cells (Figure 6C-E), although the cellular levels of 2A and 2B were significantly higher than 2C in HFF1 cells (Figure 6D), suggesting that A375 cells depended on PIP4K2C. This conclusion was confirmed by suppression of PIP4K2C protein synthesis with si*PIP4K2C*.

WX8 binds PIP4K2C with a Kd of 340 nM (Fig. S2) and inhibited enzyme activity with an IC_50_ of ~1 µM by competing with ATP binding (Figure 2D). Transient knock-down of PIP4K2C protein with si*PIP4K2C* in WX8-sensitive A375 cells inhibited cell proliferation but did not disrupt autophagy, whereas si*PIP4K2C* together with selective inhibition of PIKFYVE by 0.05 µM WX8 induced cell death (Figure 5A,C,E). In contrast, si*PIP4K2C* slightly stimulated WX8-resistant HFF1 cell proliferation, reduced SQSTM1 levels, and did not stimulate cell death in the presence of 0.05 µM WX8 (Figure 5B,D,F). Thus, inhibition of both PIKFYVE and PIP4K2C were required to arrest cell proliferation and induce cell death in PIKFYVE-dependent cells. In contrast, selective inhibition of PIP4K2C/PIP4Kγ in HEK293T cells increases basal level autophagy [45]. HEK293T cells, like HFF1 cells, are WX8-resistant [8].

Although morpholino adducts are a common feature of PIKFYVE inhibitors, they are also common to inhibitors of other phosphoinositide kinases that differ in the adducts to their central core element [60]. Therefore, differences in chemical structures not only can affect the affinity of these inhibitors for PIKFYVE, but they can also affect their affinities for secondary targets.

The primary target for YM-201636 is PIKFYVE [61,62]. However, at 1 µM YM-201636 secondary targets are the three catalytic subunits for phosphatidylinositol-4,5-bisphosphate 3-kinase (PIK3CA, B and D) [61,62]. Thus, at low concentrations YM-201636 selectively inhibits PIKFYVE, whereas at higher concentrations it will also inhibit synthesis of PtdIns(3,4,5)P_3_, which functions to activate signaling pathways required for cell growth and survival [63]. Furthermore, a mutation in *PIKFYVE* that prevents inhibition by Apilimod is ineffective against YM-201636 [10], revealing that YM-201636 inhibits PIKFYVE activity in a manner different from apilimod or WX8. Moreover, the YM-201636 analog APY0201 is strongly preferred over either YM-201636 or apilimod in treatment of multiple myeloma [28], revealing that differences in target specificity affect therapeutic potential.

Apilimod/STA-5326 [9] and its NDF analog [8] are highly specific for PIKFYVE protein *in vitro* (Fig. S2). As with WX8, low concentrations of apilimod induce ATP loss and inhibition of cell proliferation, whereas 10 to 100X higher concentrations are required to induce non-canonical apoptosis and activation of caspases 3 and 7 [9], suggesting that apilimod also has secondary targets. For example, apilimod can trigger expression of inflammatory cytokines [64], induce TFEB (transcription factor EB) to upregulate expression of genes required for autophagy and lysosomal function by migrating from the cytoplasm to the nucleus [65–67], and upregulate expression of TFEB in some cell lines but not in others [9,10,66].

The primary target for ESK981/CEP-11981 is PIKFYVE, but its secondary targets are PIP5K1A and PIP5K1C [12]. Thus, at low concentrations, ESK981 inhibits PIKFYVE, whereas at higher concentrations, both pathways for PtdIns(4,5)P_2_ synthesis are inhibited, thereby shutting down autophagy. ESK981 also inhibits kinases implicated in angiogenesis [12]. The chemical structure of ESK981 lacks any resemblance to the other three groups of PIKFYVE inhibitors (Fig. S1), suggesting that ESK981 is not a competitive inhibitor of ATP binding. Therefore, either ESK981 acts allosterically on the 240 kDa PIKFYVE protein, or it inhibits one of the other two proteins PIKFYVE-VAC14-FIG4 heterotrimer that constitutes the active form of the PIKFYVE kinase [43].

### PIKFYVE and human disease

Mutations in the PIKFYVE-VAC14-FIG4 heterotrimer that decrease cellular levels of PtdIns(3,5)P_2_ and PtdIns5P are linked to a variety of human diseases, particularly those of the nervous system [27,43] and cancer [68,69]. PIKFYVE inhibitors have therapeutic potential to eliminate PIKFYVE-dependent cancer cells, such as those in some melanoma [22] and colorectal cancers [10], as well as pluripotent cancer stem cells, such those derived from teratocarcinomas [11]. In addition, low concentrations of PIKFYVE inhibitors that disrupt lysosome homeostasis can work synergistically with other cancer sensitive inhibitors [10], thereby potentially reducing the toxicity of commonly used anti-cancer drugs such as cisplatin and etoposide by increasing efficacy with lower doses [70,71]. Successful application of PIKFYVE inhibitors will require a comprehensive understanding of the pathways regulated by these inhibitors, their secondary targets, and the ability to identify PIP5K1C phosphoinositide kinase deficient cancers. Thus, given the disparities in PIP5K1C protein levels between WX8-sensitive and resistant cells, the dependence of nonmalignant cells on PIP5K1C to maintain PtdIns(4,5)P_2_, cancers that would respond to treatment with PIKFYVE inhibitors could be identified clinically as cancers with low levels of PIP5K1C.

## Materials and methods

### Cells and cell culture

Melanoma A375 [CRL-1619], HFF1 skin fibroblasts [SCRC-1041], VeroE6 epithelial kidney [CRL-1586], U20S [HTB-96], HCT116 [CCL-247], SW480 [CCL-228], MeWo [HTB-65], G361 [CRL-1424], 293T [CRL-3216], Hs-27 [CRL-1634], and SK-MEL-28 [HTB-72] cells were from the American Type Culture Collection. Other melanoma cell lines were from [72]. HBEC lung cells were from [11]. Cells were cultured routinely in Dulbecco’s Modified Eagle’s medium supplemented with L-glutamine, 4.5 g/L glucose, sodium pyruvate, and phenol red (Corning, 10–013-CV), and normal 10% fetal bovine serum (Sigma Aldrich, F0926) at 37°C in 5% CO_2_. Cells were seeded at less than 5000 cells/cm^2^ to optimize their sensitivity to WX8 (Fig. S4). From 15 to 19 h later, the indicated compound was added in 1:1000 dilutions. Results were the same using either heat-inactivated or normal serum.

### Reagents

1 H-indole-3-carbaldehyde (4-anilino-6-[4-morpholinyl]-1,3,5-triazin-2-yl)hydrazone (WX8) was from SPECS (MLS000543798). UNC3230 was from MedChemExpress (HY-110150). ONTARGET SMART-pools and non-targeting control siRNAs were designed by Dharmacon. Cell-Titer Glo kit was from Promega Biotechnology (G7570).

### ATP assay

The concentration of WX8 required to reduce the cellular ATP level by 50% (IC_50_) was used as an indicator of the effect of WX8 on cell viability, as previously described [8]. Cells were seeded into 96-well plates (1,000 cells/well, 3125 cells/cm^2^), and either vehicle or WX8 was added the following day to give concentrations from 0 to 100 µM WX8 [8]. Cells were cultured for 4-days before quantifying total cellular ATP relative to the vehicle control using the CellTiter-Glo luminescent cell viability assay (Promega, G7572) according to the manufacturer’s instructions. The IC_50_ values for WX8 were determined for 11 different cell lines representing three different human cancers and three normal human tissues (Figure 1A, B).

### Protein immunoblotting

For detection of proteins by immunoblotting, cells were seeded in 6-well plates (5x10^4^ cells/well [5263 cells/cm^2^]) and cultured until 70–80% confluent. Cells were then trypsinized, washed with ice-cold phosphate-buffered saline (ThermoFisher, J62036.K2) and lysed with SDS-lysis buffer [11]. Immunoblotting was performed as previously described [8].

### Antibodies

Antibodies from Abcam were diluted as follows: recombinant anti-PIP5K1C (ab109192) 1:1000, PIP5K1B (ab154818) 1:1000, CHUK/IKK alpha 1:1000 and VCL/vinculin (ab219649) 1:10,000. Antibodies from Cell Signaling Technology were diluted as follows: PIP5K1A (9693) 1:1000 SQSTM1 (8025) 1:5000, LC3A/B (12,741) 1:5,000, p-RPS6 S240/244 (5364) 1:20,000, RPS6 (2217) 1:20,000, and HRP-conjugated secondary anti-rabbit (7074) 1:10,000. Antibodies from Sigma-Aldrich were PIP4K2C (SAB1407977) 1:10,000, ACTB/β-actin (A5441) 1:20,000, and anti-mouse (A4416) 1:10,000. Other antibodies were PIKFYVE (Millipore, MABS522) 1:1000

### Flow cytometry assays

ANXA5-FITC Apoptosis Detection Kit (BD Biosciences, 556,570) was used to measure cell death as described previously [11]. Cells with <2N DNA content were detected using FACSCalibur flow cytometer (BD Biosciences) according to the manufacturer instructions. Raw data were analyzed using FlowJo software.

### Autophagic flux assays

The relative differences in autophagic flux among different cell lines were determined by measuring the time required for the optimum concentration of WX8 to prevent fusion of lysosomes with autophagosomes, thereby inducing the maximum accumulation of LC3-II protein [8,31].

**Step 1**: First, the optimum concentration of WX8 required for disruption of lysosome fusion with autophagosomes was determined for each cell line. 5 × 10^4^ cells of nine different cell lines seeded per well in 6-well plate (5263 cells/cm^2^) and then cultured for 16 to 18 h. Cells were then treated either with vehicle alone (no WX8) or with concentrations of WX8 up to 10 µM for 4 h, the time required for completion of autophagy in mammalian cells [32]. Cells were then trypsinized, lysed with RIPA buffer and subjected to protein immunoblotting to determine the concentration of WX8 that induced the greatest accumulation of LC3-II protein in 4 h.

**Step 2**: Second, the optimum time required to accumulate the greatest amount of LC3-II protein in the presence of the optimum concentration of WX8 was determined relative to its vehicle control. Cells were seeded as in step-1 and then cultured for 16–18 h. Cells were then treated for 0.5 to 48 h either with vehicle alone or with their corresponding concentration of WX8 that induced the greatest accumulation of LC3-II protein in 4 h. Cells were then trypsinized, lysed in RIPA Lysis and Extraction Buffer (ThermoFisher, 89,900), and subjected to immunoblotting.

**Step 3**: Finally, each cell line was cultured with their optimum WX8 concentration and optimum time of exposure to WX8 to reveal their greatest accumulation of LC3-II protein. Cells were trypsinized, lysed with RIPA buffer and subjected to Western immunoblotting.

### RNA sequence profiling

For RNA sequencing analysis, both A375 and HFF1 cells were seeded at 2000 cells/cm^2^ for 16 to 18 h. Cells were then treated with WX8 (0.05 µM and 1 µM) or an equivalent amount of vehicle for 24 h. RNA was isolated from cells using RNeasy Mini Kit (Qiagen, NC9677589) and remnant DNA was removed with on-column DNaseI (Qiagen, 79,254) digestion. The integrity of the RNA from different samples was checked using a 2100 Bioanalyzer instrument. Samples were treated in triplicate and then libraries were prepared using e-Illumina® TruSeq®Stranded mRNA Sample Preparation Kits (Illumina, 20,020,595) to enrich poly-A containing mRNA molecules by their association with poly-T oligo attached to magnetic beads. RNA sequences were identified by NICHD Molecular Genomics Sequencing Core.

### RNA transcripts per cell

The number of transcripts of a specific gene per million total transcripts (Transcripts Per Million) was calculated from RNA-sequence profiles of melanoma A375 and HFF1 fibroblasts. These data allowed the relative abundance of specific genes to be accurately determined when compared across different cell lines.

### Identification of WX8 target proteins in situ

To provide the 50 million cells required for MS analysis, A375 cells were cultured as described in 15-cm tissue culture plates. Upon confluence, the medium was replaced with fresh medium containing 0.05 µM WX8, 1 µM WX8, or an equivalent volume of vehicle (DMSO). All treatments were performed in triplicate. Cells were incubated at 37°C for 4 h to obtain maximum cytoplasmic vacuolization, after which they were washed once with ice-cold 1X phosphate-buffered saline, scrapped off the plate, and collected by centrifugation. Cell pellets were stored at −80°C until processing.

Cells were lysed by sonication in lysis buffer (50 mM HEPES, pH 7.5, 150 mM NaCl, 0.1% Triton X-100, and Phosphatase Inhibitor Cocktail II (AG Scientific, P-1518-SOL-1VIAL). The ratio of the volume of lysis buffer to cell pellet was kept at 10:1. After lysis, the samples were cleared by centrifugation, and the supernatant collected for probe labeling. Final protein concentration in the lysate was 5.2 mg/mL.

Fifty µL of a 10X aqueous solution of the ATP probe was added to 450 µL of each sample (final concentration of the probe was 20 µM). All samples were then incubated for 10 min. Following the probe reaction samples were prepared for MS and analyzed using the standard KiNativ protocol as previously described [37,38]. A total of 312 probe-labeled kinase peptides were identified and quantified in this dataset. Results were characterized using data-dependent analysis, in which the instrument agnostically identified probe-labeled peptides. A list of probe-labeled kinase peptides was assembled consistent with active site modification (targeted analysis).

### siRNA knockdown protocol

Both WX8-sensitive (A375) and WX8-resistant (HFF1) cell lines were reverse transfected with the indicated siRNA and nt-siRNA using Lipofectamine RNAiMax (ThermoFisher, 13,778,075) following the manufacturer’s instructions . Cells were seeded at 2631 cells/cm^2^ in 24-well plates during transfection. For *PIP4K2C* and *CHUK* knockdown, 24 h post-transfection, cells were treated either with 0.05 µM WX8 or an equivalent volume of DMSO and culture continued for 48 h after which the culture medium was replaced with fresh medium plus vehicle or WX8. Cells were cultured for a total of 72 h before isolating them by trypsinization. Cells were stained with trypan blue [73] to distinguish live cells from dead cells. Cells were lysed and analyzed for the indicated protein by immunoblotting whole cell extracts [8].

### PIP5K1C gene knockout protocol

*PIP5K1C* knockout was carried out using Gene Knockout Kit v2 (Synthego) with multi-guide sgRNA’s against exon-3 of the *PIP5K1C* gene. Briefly, three different nucleotides modified sgRNA’s were mixed with purified CAS9 and cells were transfected using Lipofectamine™ CRISPRMAX™ Cas9 Transfection Reagent (ThermoFisher, CMAX00001). After 72 h, the cells were diluted so that single cells could be isolated in 96-well plates and then cultured into colonies. Individual colonies were checked for PIP5K1C expression by immunoblotting PIP5K1C protein. Selected clones with low PIP5K1C expression were then checked for ablation of the *PIP5K1C* gene using exon-specific PCR.

### Mass spectrometry of proteins in whole cell extracts

Cells were seeded at 2000 cells/cm^2^ in triplicate, cultured for 24 h, and then cultured for 16 to 18 h in the presence of either vehicle or 1 µM WX8. Cells were then lysed, and protein concentrations were measured using the “Pierce™ BCA Protein Assay Kit-Reducing Agent Compatible” (ThermoFisher, 23,252). Equal amounts (100 μg) of total protein from of each of 12 samples were processed for MS analysis by Poochon Scientific. Peptides were identified by a combination of high-performance liquid chromatography and high-resolution tandem MS. Raw data files were searched against human protein sequence databases from the UniprotKB website using the Proteome Discoverer 2.4 software (ThermoFisher, OPTON-30956) based on the SEQUEST and percolator algorithms.

### Mass ELISA of PtdIns3P, PtdIns4P and PtdIns(4,5)P_2_

Briefly, 5 × 10^5^ cells were seeded in 35 mm diameter cell culture dishes and cultured for 12 to 16 h. Cells were then cultured for 24 h with either vehicle or 1 µM WX8 and kept on ice for 5 min before total protein and lipids were collected using ice-cold 0.5 M trichloroacetic acids. For determining different phosphoinositides under *PIP4K2C* knockdown conditions, experimental conditions similar to the siRNA knockdown protocol were used. The acidic lipid was extracted from cell lysates with chloroform: methanol: 12 N HCl (40:80:1). Mixture of chloroform and 0.1 N HCl was added to the supernatant. The bottom organic phase was collected, dried, and suspended in 200 µL of solvent unique for different phosphoinositide ELISA. All lipid samples are sonicated at room temperature in a Diagenode’s Bioruptor 300 (high power setting, 30s ON, 30s OFF) for 10 min before being subjected to Mass ELISA assays for PtdIns3P (Echelon Biosciences, K-3300), PtdIns4P (K-4000E) and PtdIns(4,5)P_2_ (K4500) according to the manufacturer’s instructions. All the ELISA assays were performed twice and the concentration of different phosphoinositides was calculated from the standards generated using non-linear regression analysis with GraphPad Prism software. Sigmoidal dose-response with variable slope curve analysis (four-parameter, 4PL curve fit) was utilized.

### PIP5K1C overexpression

Two isoforms of *PIP5K1C* (PIP5K1C-1 [NM_001195733.2] and PIP5K1C-2 [NM_012398.3]) were detected in HFF1 cells using isoform-specific primer pairs from the cDNA made from HFF1 RNA. Isoform-3 (NM_001300849.2) was not detected. PCR-amplified *PIP5K1C-1* and *PIP5K1C-2* DNA were then cloned into the pcDNA™3.4 TOPO™ TA vector (ThermoFisher, A14697) following the manufacturers’ protocol. Melanoma A375 cells were transfected, and clones with the correct orientation of amplified PCR were selected that expressed the PIP5K1C isoform protein.

To make overexpressing PIP5K1C cell lines, A375 cells were plated in 24-well plates (10^5^ cells/well) and then transfected independently with PIP5K1C-1, PIP5K1C-2, or pCMV-GFP expression vectors. At 24 h post-transfection, cells were selected for resistance in 1000 µg/mL Geneticin/G418 Sulfate (Thermo Fisher, 10,131,035), which killed all the untransfected cells. After 14 days of G418 selection, cells expressing the PIP5K1C isoform were obtained based on the green fluorescent protein signal from cells in pCMV-GFP transfected wells. These cells were then tested for their sensitivity to increasing concentrations of WX8.

### Statistical calculations

All statistical analyses were performed using Prism software (GraphPad). Figure legends indicate the number of times an experiment was repeated. Either the standard deviation or the standard error of the mean is indicated in each bar and line graph. P-values were determined by Student’s unpaired two-tailed *t*-test.

### Data sharing

MS data are available upon request.

## Supplementary Material

Supplemental Material
