# Supplementary material for: PIP5K1C phosphoinositide kinase deficiency distinguishes PIKFYVE-dependent cancer cells from non-malignant cells
Source: Autophagy. 2023 Mar 22;19(9):2464–84. doi: 10.1080/15548627.2023.2182594 (PMC10392749; doi:10.1080/15548627.2023.2182594)
Supplement: Supplemental Material [file KAUP_A_2182594_SM3428.zip › Editable MS Word copy of Supplementary Figures 02142023.docx]

**
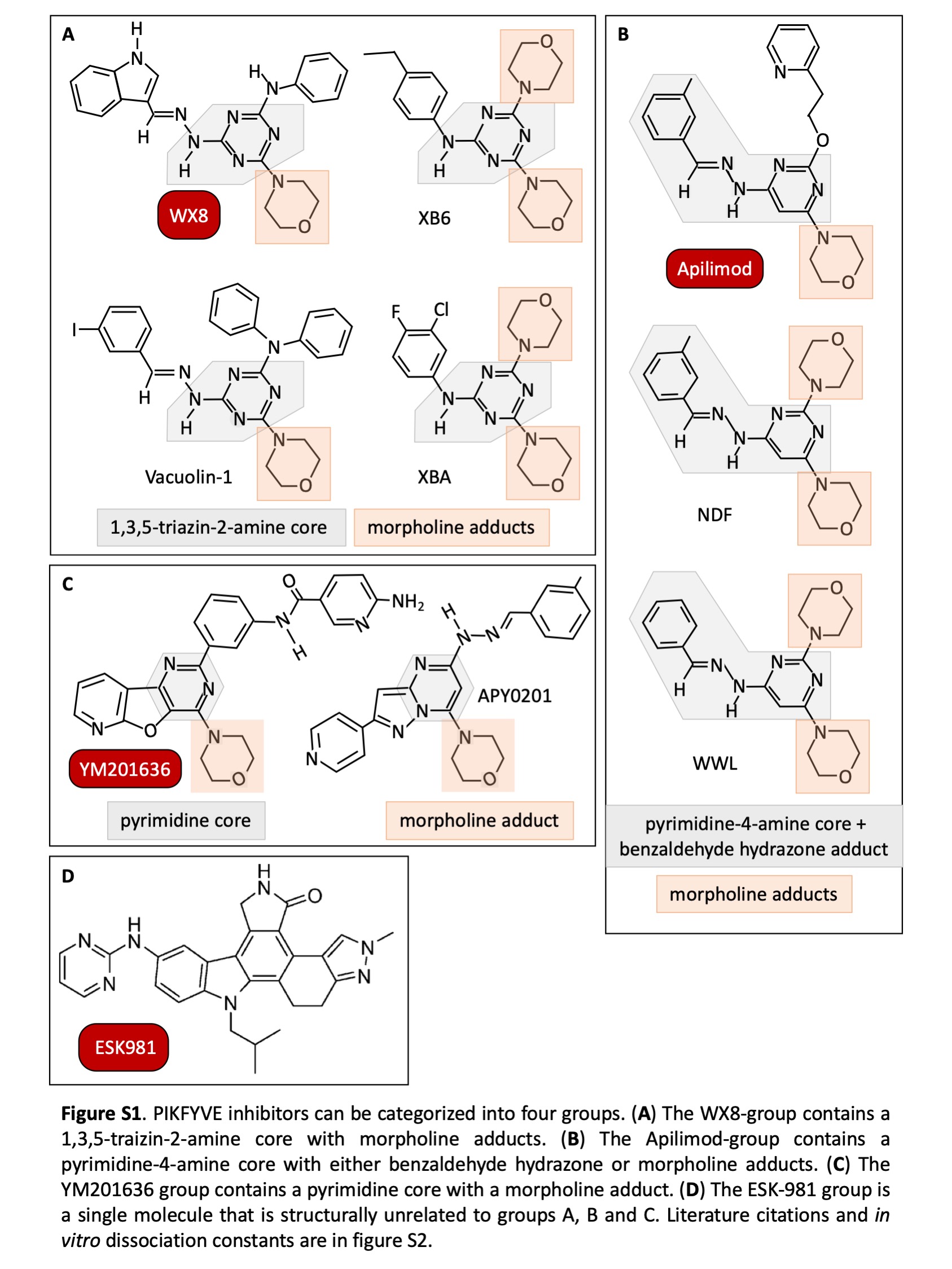
**

**Figure S1**. PIKFYVE inhibitors can be categorized into four groups. (**A**) The WX8-group contains a 1,3,5-traizin-2-amine core with morpholine adducts. (**B**) The Apilimod-group contains a pyrimidine-4-amine core with either benzaldehyde hydrazone or morpholine adducts. (**C**) The YM201636 group contains a pyrimidine core with a morpholine adduct. (**D**) The ESK-981 group is a single molecule that is structurally unrelated to groups A, B and C. Literature citations and *in vitro* dissociation constants are in figure S2


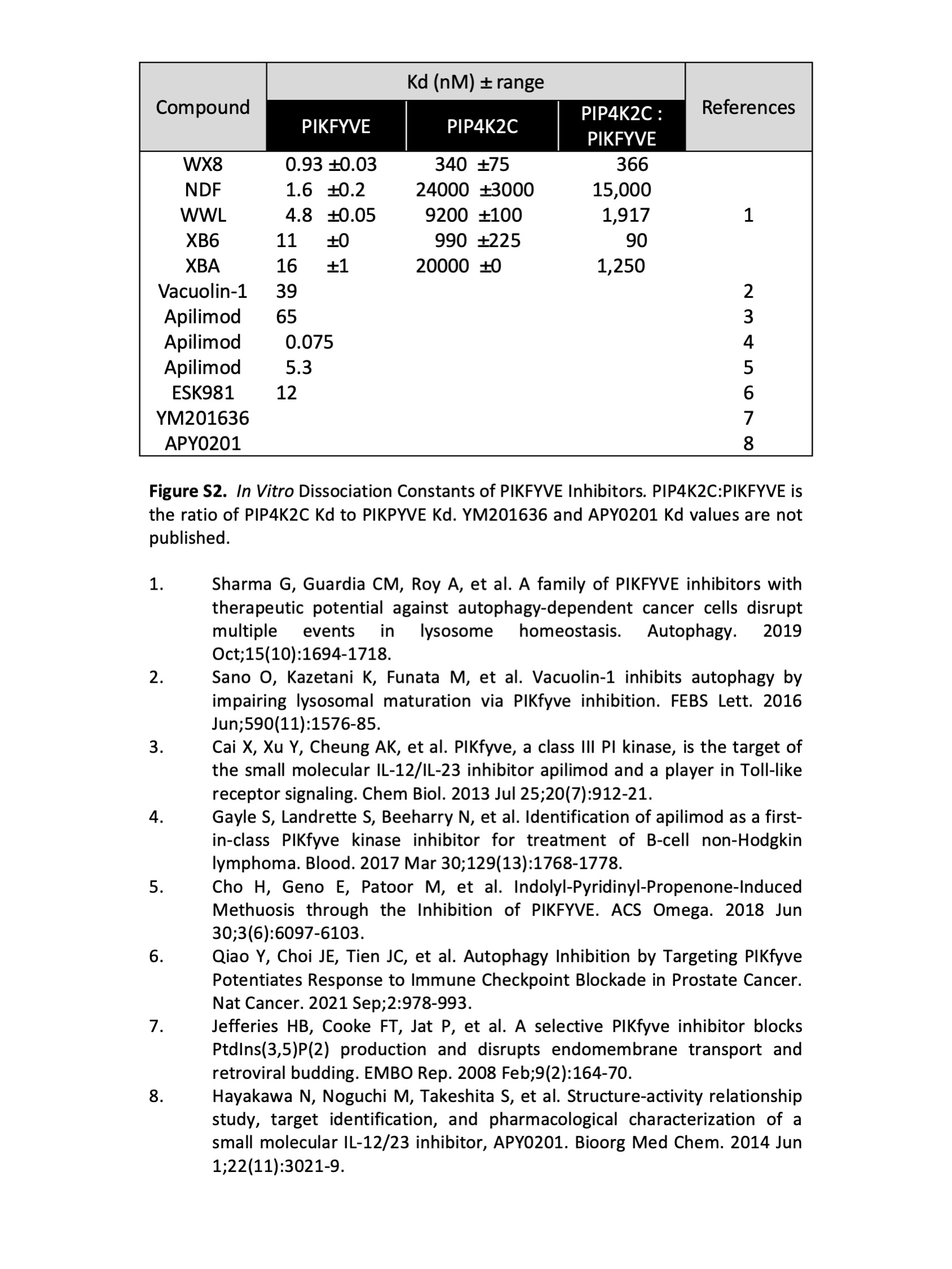


**Figure S2.** *In Vitro* Dissociation Constants of PIKFYVE Inhibitors*.* PIP4K2C:PIKFYVE is the ratio of PIP4K2C Kd to PIKPYVE Kd. YM201636 and APY0201 Kd values are not published.

1. Sharma G, Guardia CM, Roy A, et al. A family of PIKFYVE inhibitors with therapeutic potential against autophagy-dependent cancer cells disrupt multiple events in lysosome homeostasis. Autophagy. 2019 Oct;15(10):1694-1718.

2. Sano O, Kazetani K, Funata M, et al. Vacuolin-1 inhibits autophagy by impairing lysosomal maturation via PIKfyve inhibition. FEBS Lett. 2016 Jun;590(11):1576-85.

3. Cai X, Xu Y, Cheung AK, et al. PIKfyve, a class III PI kinase, is the target of the small molecular IL‑12/IL-23 inhibitor apilimod and a player in Toll-like receptor signaling. Chem Biol. 2013 Jul 25;20(7):912-21.

4. Gayle S, Landrette S, Beeharry N, et al. Identification of apilimod as a first-in-class PIKfyve kinase inhibitor for treatment of B-cell non-Hodgkin lymphoma. Blood. 2017 Mar 30;129(13):1768-1778.

5. Cho H, Geno E, Patoor M, et al. Indolyl-Pyridinyl-Propenone-Induced Methuosis through the Inhibition of PIKFYVE. ACS Omega. 2018 Jun 30;3(6):6097-6103.

6. Qiao Y, Choi JE, Tien JC, et al. Autophagy Inhibition by Targeting PIKfyve Potentiates Response to Immune Checkpoint Blockade in Prostate Cancer. Nat Cancer. 2021 Sep;2:978-993.

7. Jefferies HB, Cooke FT, Jat P, et al. A selective PIKfyve inhibitor blocks PtdIns(3,5)P(2) production and disrupts endomembrane transport and retroviral budding. EMBO Rep. 2008 Feb;9(2):164-70.

8. Hayakawa N, Noguchi M, Takeshita S, et al. Structure-activity relationship study, target identification, and pharmacological characterization of a small molecular IL-12/23 inhibitor, APY0201. Bioorg Med Chem. 2014 Jun 1;22(11):3021-9.

**
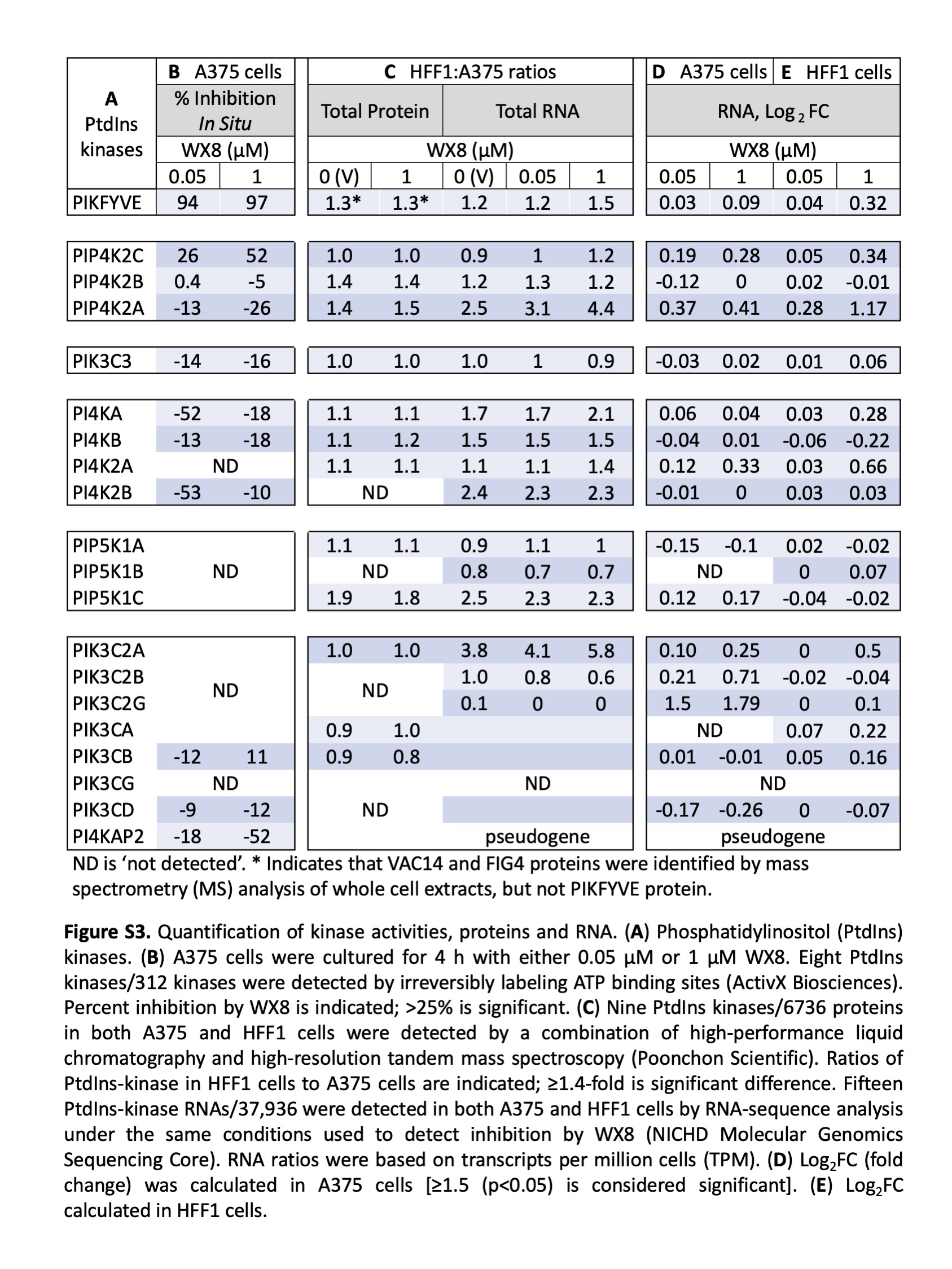
**

**Figure S3.** Quantification of kinase activities, proteins and RNA. (**A**) Phosphatidylinositol (PtdIns) kinases. (**B**) A375 cells were cultured for 4 h with either 0.05 µM or 1 µM WX8. Eight PtdIns kinases/312 kinases were detected by irreversibly labeling ATP binding sites (ActivX Biosciences). Percent inhibition by WX8 is indicated; >25% is significant. (**C**) Nine PtdIns kinases/6736 proteins in both A375 and HFF1 cells were detected by a combination of high‑performance liquid chromatography and high‑resolution tandem mass spectroscopy (Poonchon Scientific). Ratios of PtdIns-kinase in HFF1 cells to A375 cells are indicated; ≥1.4-fold is significant difference. Fifteen PtdIns-kinase RNAs/37,936 were detected in both A375 and HFF1 cells by RNA-sequence analysis under the same conditions used to detect inhibition by WX8 (NICHD Molecular Genomics Sequencing Core). RNA ratios were based on transcripts per million cells (TPM). (**D**) Log_2_FC (fold change) was calculated in A375 cells [≥1.5 (p<0.05) is considered significant]. (**E**) Log_2_FC calculated in HFF1 cells.


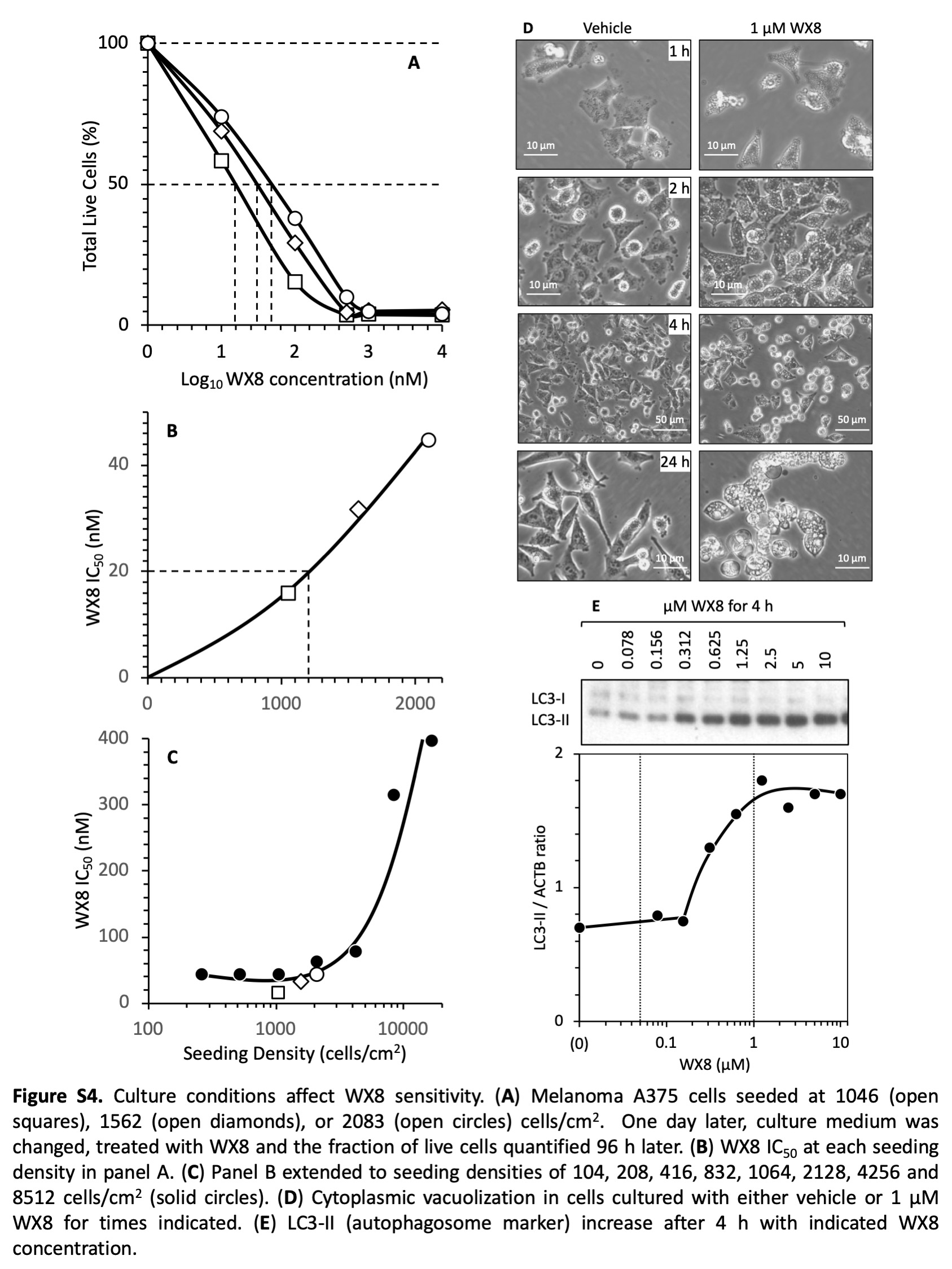


**Figure S4.** Culture conditions affect WX8 sensitivity. (**A**) Melanoma A375 cells seeded at 1046 (open squares), 1562 (open diamonds), or 2083 (open circles) cells/cm^2^. One day later, culture medium was changed, treated with WX8 and the fraction of live cells quantified 96 h later. (**B**) WX8 IC_50_ at each seeding density in panel A. (**C**) Panel B extended to seeding densities of 104, 208, 416, 832, 1064, 2128, 4256 and 8512 cells/cm^2^ (solid circles). (**D**) Cytoplasmic vacuolization in cells cultured with either vehicle or 1 µM WX8 for times indicated. (**E**) LC3-II (autophagosome marker) increases after 4 h with indicated WX8 concentration.


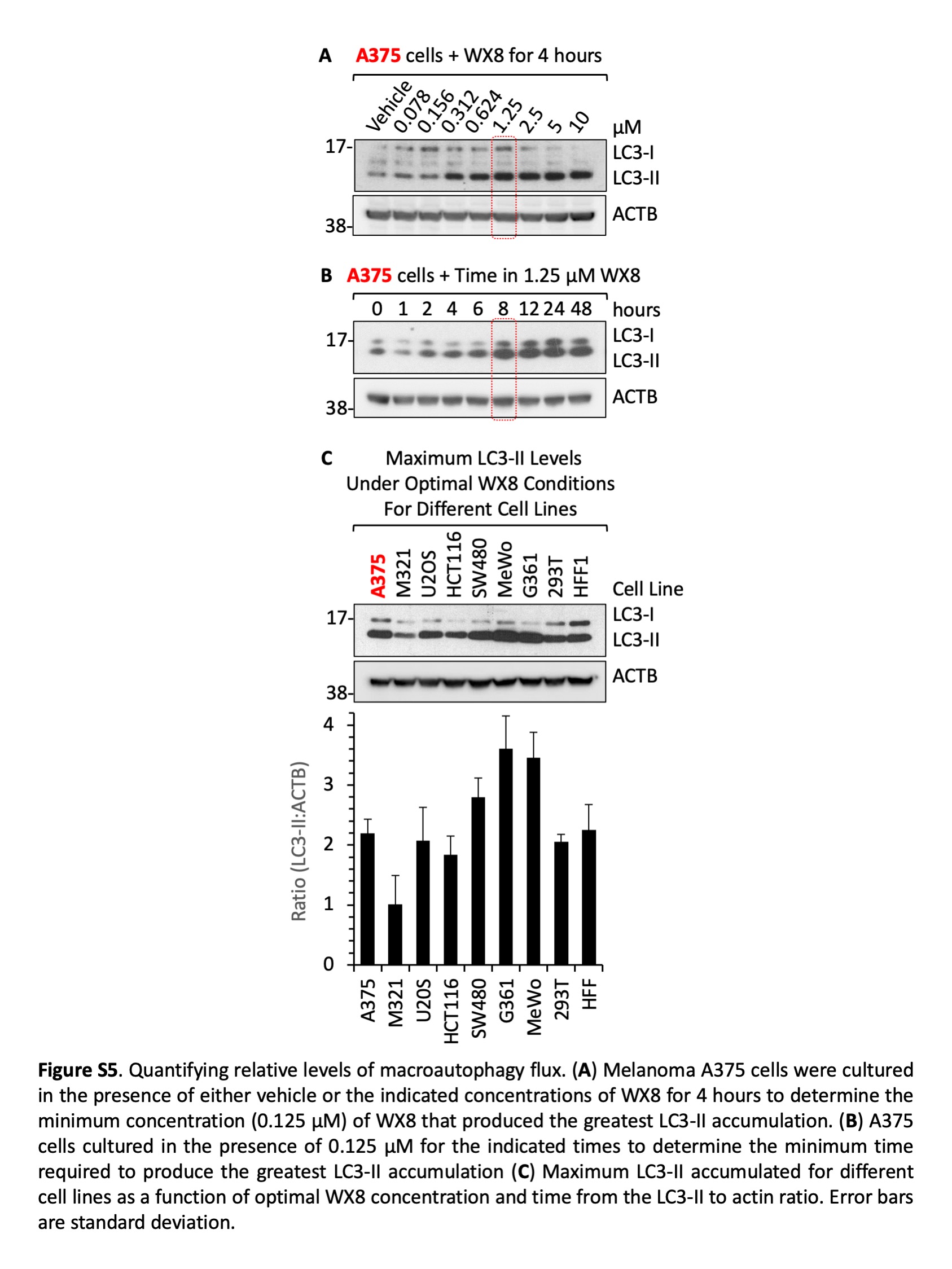


**Figure S5**. Quantifying relative levels of macroautophagy flux. (**A**) Melanoma A375 cells were cultured in the presence of either vehicle or the indicated concentrations of WX8 for 4 hours to determine the minimum concentration (0.125 µM) of WX8 that produced the greatest LC3-II accumulation. (**B**) A375 cells cultured in the presence of 0.125 µM for the indicated times to determine the minimum time required to produce the greatest LC3-II accumulation (**C**) Maximum LC3-II accumulated for different cell lines as a function of optimal WX8 concentration and time from the LC3-II to actin ratio. Error bars are standard deviation.


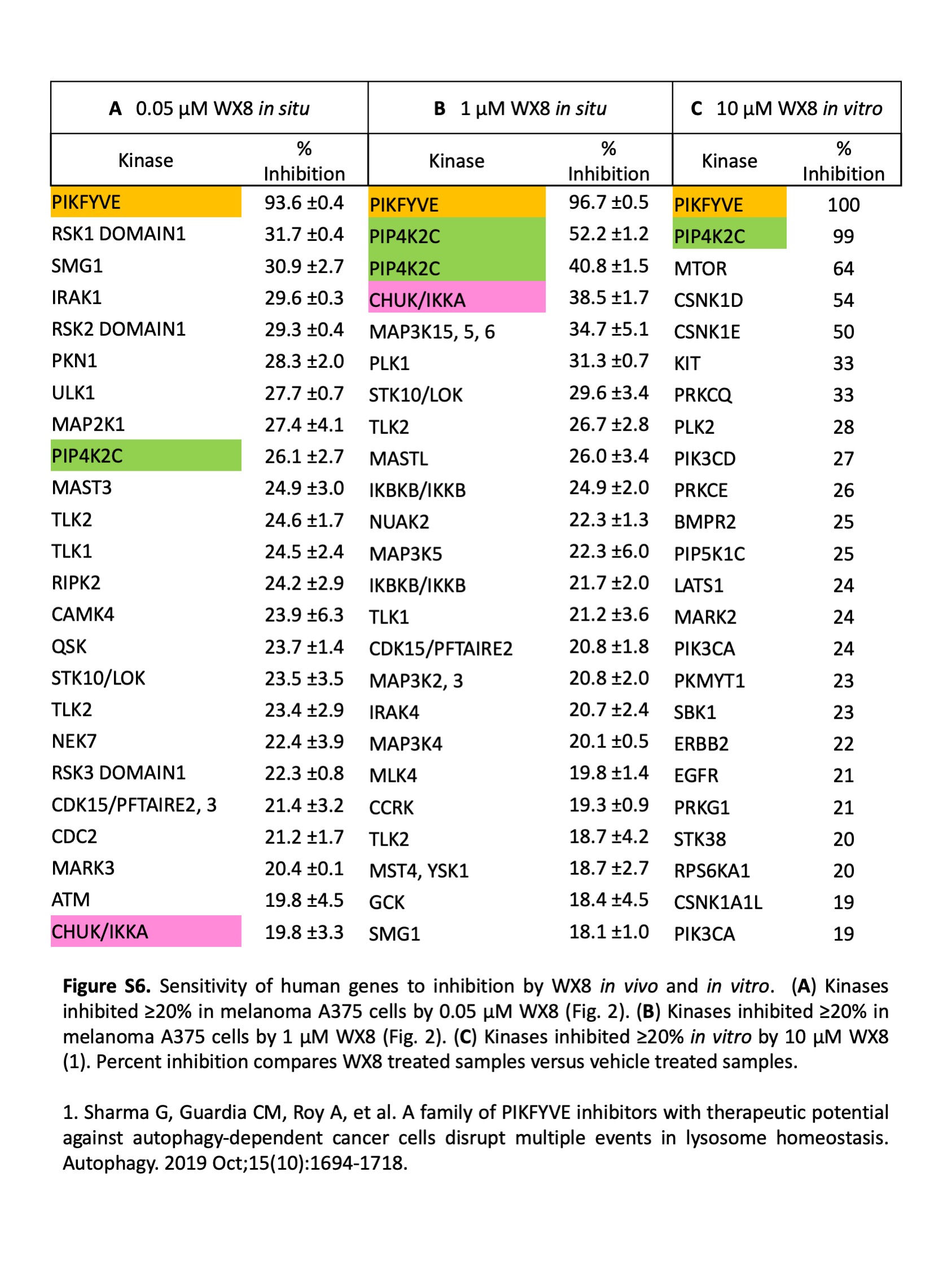


**Figure S6.** Sensitivity of human genes to inhibition by WX8 *in vivo* and *in vitro*. (**A**) Kinases inhibited ≥20% in melanoma A375 cells by 0.05 µM WX8 (Fig. 2). (**B**) Kinases inhibited ≥20% in melanoma A375 cells by 1 µM WX8 (Fig. 2). (**C**) Kinases inhibited ≥20% *in vitro* by 10 µM WX8 (1). Percent inhibition compares WX8 treated samples versus vehicle treated samples.

1. Sharma G, Guardia CM, Roy A, et al. A family of PIKFYVE inhibitors with therapeutic potential against autophagy-dependent cancer cells disrupt multiple events in lysosome homeostasis. Autophagy. 2019 Oct;15(10):1694-1718.


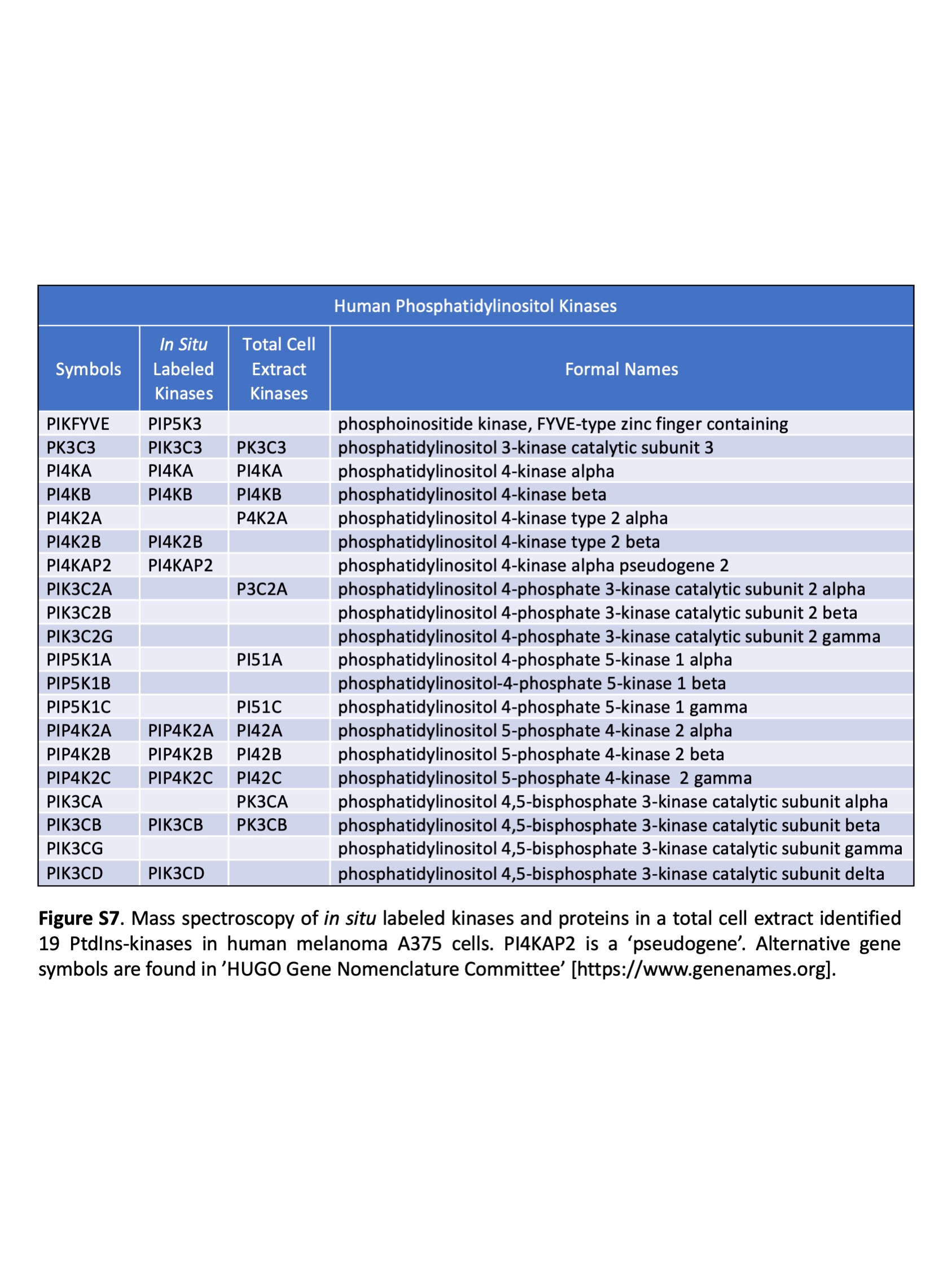


**Figure** **S7**. Mass spectroscopy of *in situ* labeled kinases and proteins in a total cell extract identified 19 PtdIns-kinases in human melanoma A375 cells. PI4KAP2 is a ‘pseudogene’. Alternative gene symbols are found in ’HUGO Gene Nomenclature Committee’ [https://www.genenames.org].

**
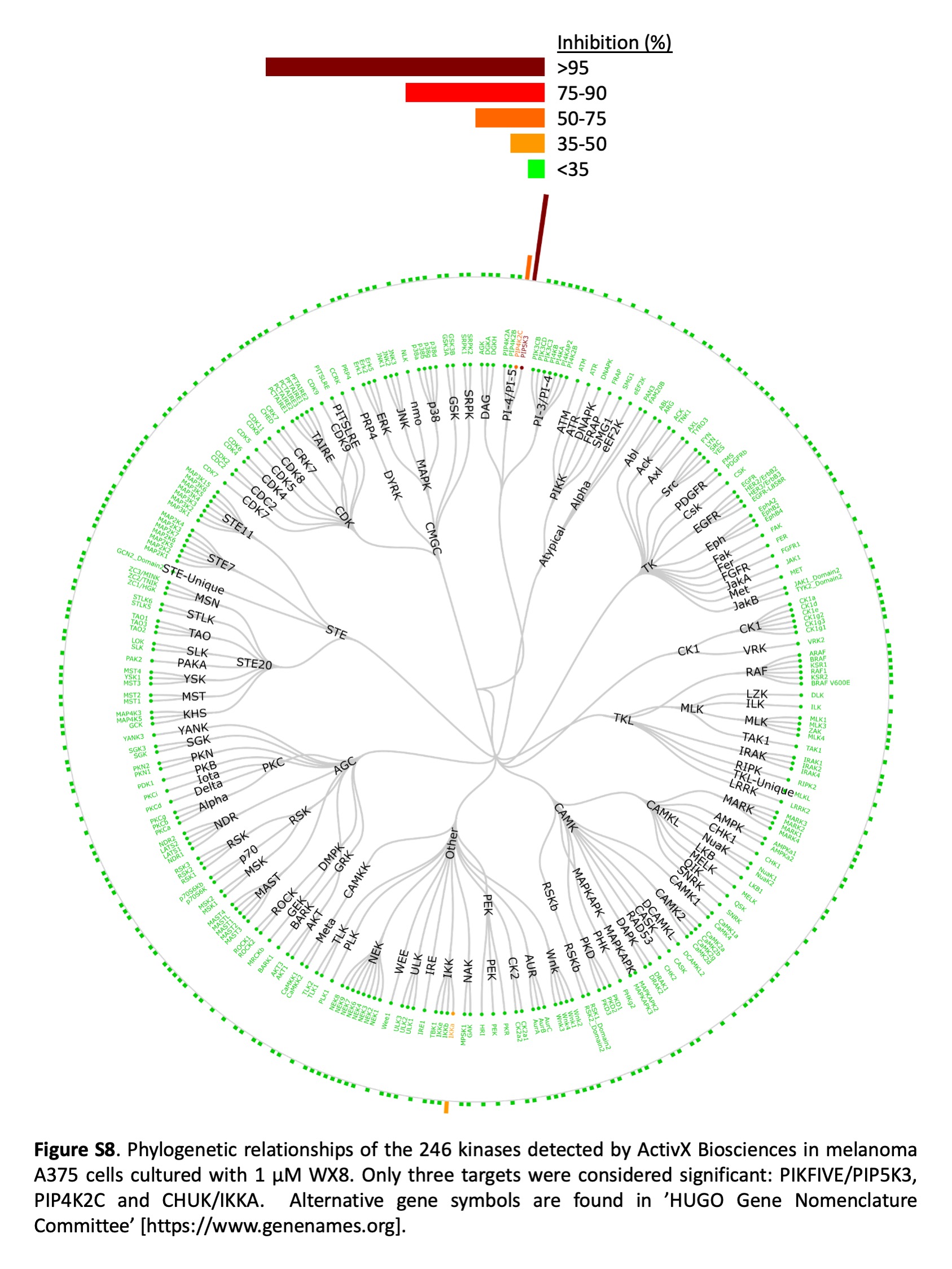
**

**Figure S8**. Phylogenetic relationships of the 246 kinases detected by ActivX Biosciences in melanoma A375 cells cultured with 1 µM WX8. Only three targets were considered significant: PIKFIVE/PIP5K3, PIP4K2C and CHUK/IKKA. Alternative gene symbols are found in ’HUGO Gene Nomenclature Committee’ [https://www.genenames.org].


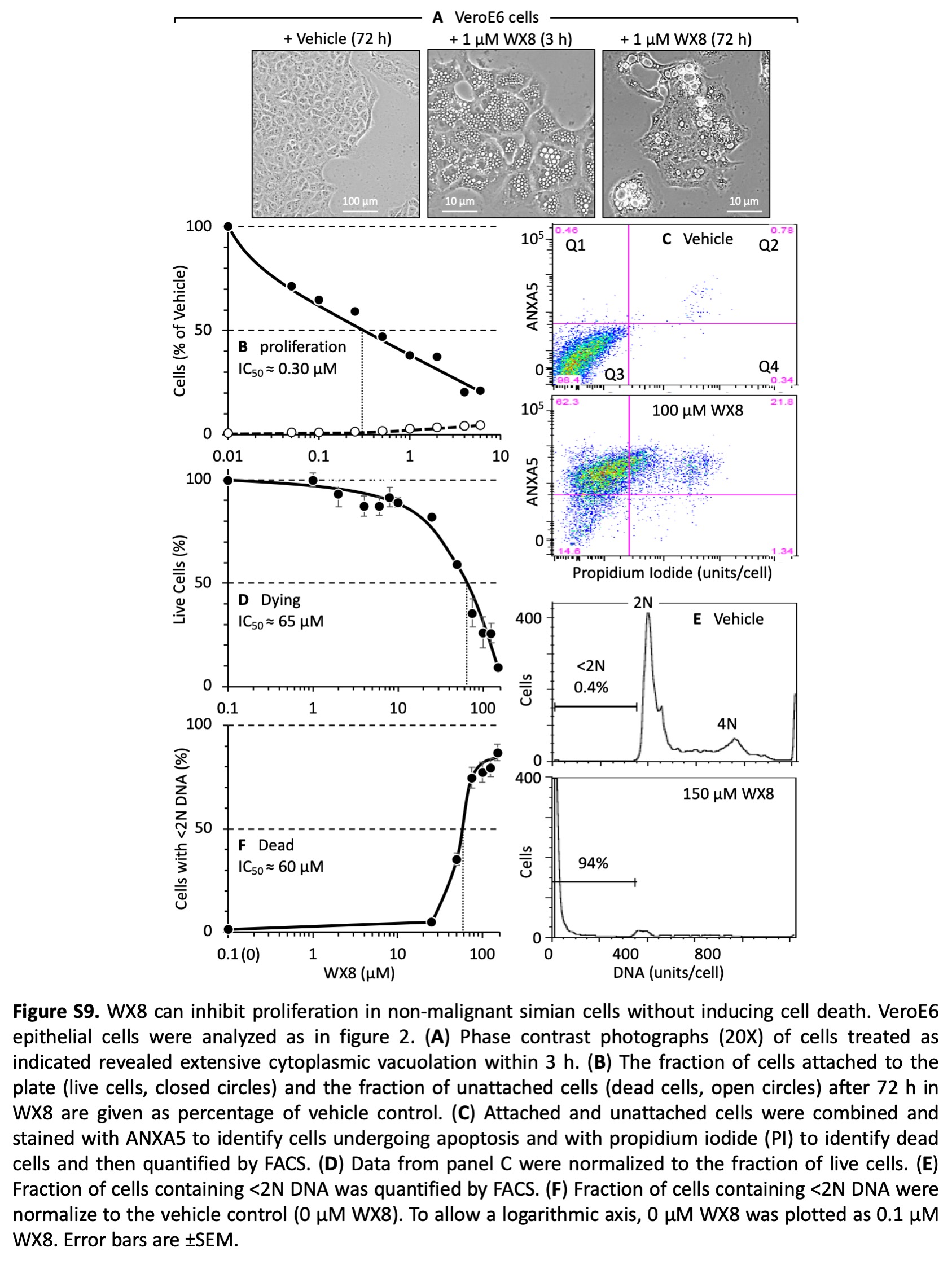


**Figure S9.** WX8 can inhibit proliferation in non-malignant simian cells without inducing cell death. VeroE6 epithelial cells were analyzed as in figure 2. (**A**) Phase contrast photographs (20X) of cells treated as indicated revealed extensive cytoplasmic vacuolation within 3 h. (**B**) The fraction of cells attached to the plate (live cells, closed circles) and the fraction of unattached cells (dead cells, open circles) after 72 h in WX8 are given as percentage of vehicle control. (**C**) Attached and unattached cells were combined and stained with ANXA5 to identify cells undergoing apoptosis and with propidium iodide (PI) to identify dead cells and then quantified by FACS. (**D**) Data from panel C were normalized to the fraction of live cells. (**E**) Fraction of cells containing <2N DNA was quantified by FACS. (**F**) Fraction of cells containing <2N DNA were normalize to the vehicle control (0 µM WX8). To allow a logarithmic axis, 0 µM WX8 was plotted as 0.1 µM WX8. Error bars are ±SEM.
